# Small area disease mapping of cancer incidence in British Columbia using Bayesian spatial models and the smallareamapp R Package

**DOI:** 10.3389/fonc.2022.833265

**Published:** 2022-10-19

**Authors:** Jonathan Simkin, Trevor J. B. Dummer, Anders C. Erickson, Michael C. Otterstatter, Ryan R. Woods, Gina Ogilvie

**Affiliations:** ^1^ Cancer Control Research, BC Cancer, Provincial Health Services Authority, Vancouver, BC, Canada; ^2^ School of Population and Public Health, Faculty of Medicine, University of British Columbia, Vancouver, BC, Canada; ^3^ Office of the Provincial Health Officer, Government of British Columbia, Victoria, BC, Canada; ^4^ British Columbia Centre for Disease Control, Vancouver, BC, Canada; ^5^ Faculty of Health Sciences, Simon Fraser University, Burnaby, BC, Canada; ^6^ Women’s Health Research Institute, BC Women’s Hospital + Health Centre, Vancouver, BC, Canada

**Keywords:** cancer surveillance, epidemiology, geospatial, disease mapping, spatial autoregressive analysis, BYM, Shiny, INLA

## Abstract

**Introduction:**

There is an increasing interest in small area analyses in cancer surveillance; however, technical capacity is limited and accessible analytical approaches remain to be determined. This study demonstrates an accessible approach for small area cancer risk estimation using Bayesian hierarchical models and data visualization through the smallareamapp R package.

**Materials and methods:**

Incident lung (N = 26,448), female breast (N = 28,466), cervical (N = 1,478), and colorectal (N = 25,457) cancers diagnosed among British Columbia (BC) residents between 2011 and 2018 were obtained from the BC Cancer Registry. Indirect age-standardization was used to derive age-adjusted expected counts and standardized incidence ratios (SIRs) relative to provincial rates. Moran’s *I* was used to assess the strength and direction of spatial autocorrelation. A modified Besag, York and Mollie model (BYM2) was used for model incidence counts to calculate posterior median relative risks (RR) by Community Health Service Areas (CHSA; N = 218), adjusting for spatial dependencies. Integrated Nested Laplace Approximation (INLA) was used for Bayesian model implementation. Areas with exceedance probabilities (above a threshold RR = 1.1) greater or equal to 80% were considered to have an elevated risk. The posterior median and 95% credible intervals (CrI) for the spatially structured effect were reported. Predictive posterior checks were conducted through predictive integral transformation values and observed versus fitted values.

**Results:**

The proportion of variance in the RR explained by a spatial effect ranged from 4.4% (male colorectal) to 19.2% (female breast). Lung cancer showed the greatest number of CHSAs with elevated risk (N_women_ = 50/218, N_men_ = 44/218), representing 2357 total excess cases. The largest lung cancer RRs were 1.67 (95% CrI = 1.06–2.50; exceedance probability = 96%; cases = 13) among women and 2.49 (95% CrI = 2.14–2.88; exceedance probability = 100%; cases = 174) among men. Areas with small population sizes and extreme SIRs were generally smoothed towards the null (RR = 1.0).

**Discussion:**

We present a ready-to-use approach for small area cancer risk estimation and disease mapping using BYM2 and exceedance probabilities. We developed the smallareamapp R package, which provides a user-friendly interface through an R-Shiny application, for epidemiologists and surveillance experts to examine geographic variation in risk. These methods and tools can be used to estimate risk, generate hypotheses, and examine ecologic associations while adjusting for spatial dependency.

## Introduction

Spatial epidemiology is an integral part of cancer surveillance and research. Disease mapping, a common spatial methodology, provides a visual summary of geographic information and has a long history in population oncology (1, 2). In the 1930s, Stocks et al. reported cancer mortality maps by counties in England and Wales (3–5). Today, cancer atlases are fundamental to international cancer surveillance, including the Cancer in Five Continents monograph (6), and web-based interactive platforms such as Global Cancer Observatory (7) and the National Cancer Institute’s Cancer Atlas (8).

Traditional geographic analyses are often based on relatively broad geographic areas (2, 9). While these are useful for high-level surveillance and planning purposes, small area analyses present new opportunities to understand local disease patterns (2, 10). Compared to broad area studies, small area analyses are better approaches for detecting environmental effects when exposures are highly localized (2, 10). Risk factors and social and behavioral determinants of health are often more similar within smaller geographic units (11). Small area studies are also less susceptible to ecological bias (2, 10, 11). In terms of surveillance, true excess disease risk may be overlooked when using broad regional areas (2, 12). The desire for small area analyses has been longstanding (2, 13); however, barriers to widespread adoption remain, including technically sophisticated methodologies which are not easily implemented and the need to protect personal privacy. Further, technical challenges to small area analyses in general are unstable and unreliable risk estimates for areas with small populations (2, 13–16).

Additionally, people and communities are often clustered geographically, which may have important influences on disease rates (2). When data are clustered, traditional statistical methods relying on independent observations are not appropriate (17). Recent advances in data availability, development of analytical tools, methodological approaches, and technological capacity in geographic information systems have enabled investigators to examine variations in disease among small areas (2, 9, 14, 15, 18–20). In particular, Bayesian hierarchical models are often used for spatially smoothing risk estimates, increasing stability, accounting for spatial dependence, and protecting confidentiality (2, 9, 21).

Population-based cancer registries are large georeferenced datasets that allow for small area analyses. Small area analyses present new opportunities in population oncology research and surveillance; however, technical capacity is limited and accessible analytical approaches and tools remain to be determined. Using data from the British Columbia (BC) Cancer Registry (BCCR), the purpose of this study was to examine geographic variation of cancer incidence at a small area level using Bayesian hierarchical models, and demonstrate how this can be done through an accessible analytical approach based on our *smallareamapp* R package (22).

## Materials and methods

### Study setting

BC is Canada’s western-most province with a population of roughly 5.1 million people (23). BC health services are provisioned according to various health administrative areas, the largest consisting of five regional health authorities (HA). Nested within HAs are 218 Community Health Service Areas (CHSA) (24), the geographical unit of this study. The median CHSA area was 83.1 km^2^ (range = 0.92–132,241 km^2^), and the median CHSA population was 17,754 persons (range = 421–81,414 persons).

### Data sources and geocoding

New cases of lung (N = 26,448), female breast (N = 28,466), cervical (N = 1,478), and colorectal (N=25,457) cancers diagnosed among BC residents between 2011 and 2018 were obtained from the BCCR. Cancer types were classified following definitions from the Canadian Cancer Statistics publication (25). The Statistics Canada Postal Code Conversion File Plus (26) was used to assign spatial locations (i.e. longitude-latitude) to cases from six-digit postal codes by year of diagnosis. Records with non-valid, non-residential, or missing postal codes were excluded (N = 1,792 or 2.2%). This included postal codes that could not be matched, those that are only linked to a post office location and for which census location data were not available, and those that indicate a non-residential address (e.g. a commercial or institutional building). More information on these linkage errors are described in the PCCF+ user guide (26).

Records were linked to CHSAs by mapping point locations to the CHSA boundary map (24). After consultation with the First Nations Health Authority (27), data for CHSAs with a population greater than 25% of Indigenous people and/or with an Indigenous name were suppressed.

### Statistical analysis

Geographical variation in cancer risk by CHSA was examined through the following approach:

1. Observed counts, age-adjusted expected counts, and standardized incidence ratios (SIR);
2. Direction and strength of spatial autocorrelation in SIRs through Moran’s *I* statistic;
3. Posterior median of relative risks (RR) derived from Bayesian Poisson spatial modeling of observed counts relative to age-adjusted expected counts, adjusting for spatial dependence;
4. Uncertainty estimates through 95% credible intervals (CrI) and exceedance probabilities from our Bayesian model;
5. The posterior median of our model’s spatially structured effect (the proportion of variance in the modeled RRs explained by spatially structured correlations);
6. Posterior predictive checks were provided in supplementary materials. This model validation step included plotting predictive integral transformed (PIT) values (i.e. leave one out validation), plotting fitted and observed values, and Pearson residuals.

The indirect method (28) was used to calculate age-adjusted expected counts and SIRs by CHSA and sex relative to provincial age-specific cancer incidence rates. To derive SIRs, 19 five-year age groups (e.g. 0–4 years, 5–9 years … 90+ years) were used.

The modified Besag, York, and Mollie model (29) (BYM2) was used to model incident count data and calculate RRs by CHSA, adjusting for spatial correlations between neighboring areas. A queen’s contiguity matrix was used to define adjacency and spatial weights. Contiguity-based matrices define neighboring units as those that share a common border. The most basic contiguity matrix is a rook’s matrix, in which neighbors are defined as spatial units that share a common edge. The queen’s matrix is more encompassing and neighbors include those that share a common edge or vertex. The BYM2 model is a re-parameterization of the original Besag, York and Mollie model (BYM) (30) based on a generalized linear Poisson model including a spatially structured random effect and an unstructured random effect (independent and identically distributed Gaussian random effect) (30). In BYM2, the spatially structured and unstructured terms are scaled (i.e. standardized to have variance equal to one). BYM2 has two hyperparameters, a precision and mixing parameter. The precision parameter controls the variability explained by a spatial effect (29). The mixing parameter distributes existing variability between unstructured and structured model components (29). More detailed information on the BYM2 model is provided in the **Supplementary Material (Appendix A)**.

We follow Riebler et al. (29) for prior specification and used penalized complexity (PC) priors (29, 31). With PC priors, the model shrinks towards a constant risk surface when disease risk is spatially unstructured (29, 31) (i.e., no spatial dependence). The mixing parameter prior was set to (U = 0.5,α = 2/3), which assumes the unstructured random effect accounts for more of the variability than the spatially structured effect (29, 31, 32). The precision parameter was set to (U = 0.2/0.31, α = 0.01) (31). Integrated Nested Laplace Approximation (INLA) was used for Bayesian model implementation (33, 34). More programmatic information on specifying the model, prior, and correlation structure can be found on the smallareamapp GitHub repository (22), Riebler et al. (**Appendix B**) (31), or the R-INLA package website (33, 34).

Uncertainty estimates for posterior median RRs were quantified with equal tail 95% credible intervals (CrI). Exceedance probabilities were calculated using a default threshold of 10% elevated risk (RR = 1.1). Following Richardson et al., Saint-Jacques et al., and Holowaty et al., an exceedance probability equal to or greater than 80% was considered to show elevated risk (10, 19, 35). Posterior distributions of the spatial effect were right skewed and uncertainty was reported with 95% highest posterior density CrIs.

Posterior predictive checks included plotting PIT values for each observation on a log scale. A uniform distribution is expected; otherwise, a lack of model fit is indicated. The observed (SIRs) and fitted values (RRs) were plotted, as well as the observed and fitted counts.

Various posterior predictive checks were performed. Conditional predictive ordinate (CPO) and PIT values were checked for failure. Failed values were recomputed by removing a given data point and re-fitting the model to predict that data point. There were no failures among recomputed CPO/PIT values. The R-INLA package modifies PIT values for discrete responses with the following calculation: pit[i] – 0.5*cpo[i], which is discussed further by Schrodle et al. (36). The modified PIT was plotted for each observation on a logit scale (**Supplementary Figures 1B**–**4B**) and also presented in a histogram (**Supplementary Figure 5**). Deviations from uniformity indicate that model deficiencies might be present (36). Pearson residuals were also estimated following code provided by the R-INLA team (37) (**Supplementary Figure 6**). The observed SIRs and fitted values RRs were plotted to assess spatial smoothing (**Supplementary Figures 1A**–**4A**). The observed and fitted counts were plotted to assess posterior predictive performance (**Supplementary Figure 7**).

Prior to small area risk estimation, global Moran’s *I* statistic was calculated to understand whether risk estimates were clustered or not. We tested the null hypothesis of no spatial autocorrelation (SIRs are randomly distributed) with the Monte Carlo simulation method (N = 999 simulations). Statistically significant positive spatial autocorrelation (SIRs are clustered) was determined using a p-value less than 0.05.

All analyses were performed using R version 4.1.0 (38) and RStudio (39). Indirect age-standardization was performed using the epitools R-package (28). In relation to the First Nations OCAP^®^ principles (40), the First Nations Health Authority of BC recommended suppressing the modeled relative risks for 23 CHSAs with an Indigenous name or with a majority population of Indigenous people. Data for suppressed areas were included in all analyses to ensure neighboring regions could draw from the corresponding information. Modeled relative risks from the 23 CHSAs were only suppressed from final tables and disease maps. All spatial analyses presented were carried out using the R-package s*mallareamapp* (22) that we developed and includes dependency R-packages sf (41), raster (42), tmap (43), and R-INLA (33, 34). The *smallareamapp* package hosts an R Shiny application that provides a user-friendly interface for model fitting, implementation using INLA, and diagnostics, as well tailoring of output metrics (e.g. relative risk, exceedance probabilities). Maps, table summaries and model summaries can be created and exported from the application directly. The maps presented here were generated in R using the tmap and grid R packages based on data summaries exported from *smallareamapp*. An overview and walkthrough of the *smallareamapp* package can be found in **Appendix B** (additional file) and the Github repository (22).

## Results

### Lung cancer

The Moran’s *I* statistic among female lung cancer SIRs was 0.259 (p = 0.001) and 0.347 (p = 0.001) among men, indicating significant clustering. Among women, observed cases of lung cancer by CHSA ranged from 0 to 254 cases. Women’s SIRs ranged from 0.35 to 3.63 among non-zero count areas. Women’s RRs ranged from 0.53 to 1.63. Among men, observed cases by CHSA ranged from 2 to 218 cases. Men’s SIRs ranged from 0.29 to 2.69 among non-zero count areas. Men’s RRs ranged from 0.49 to 2.49.

**Figure 1:**
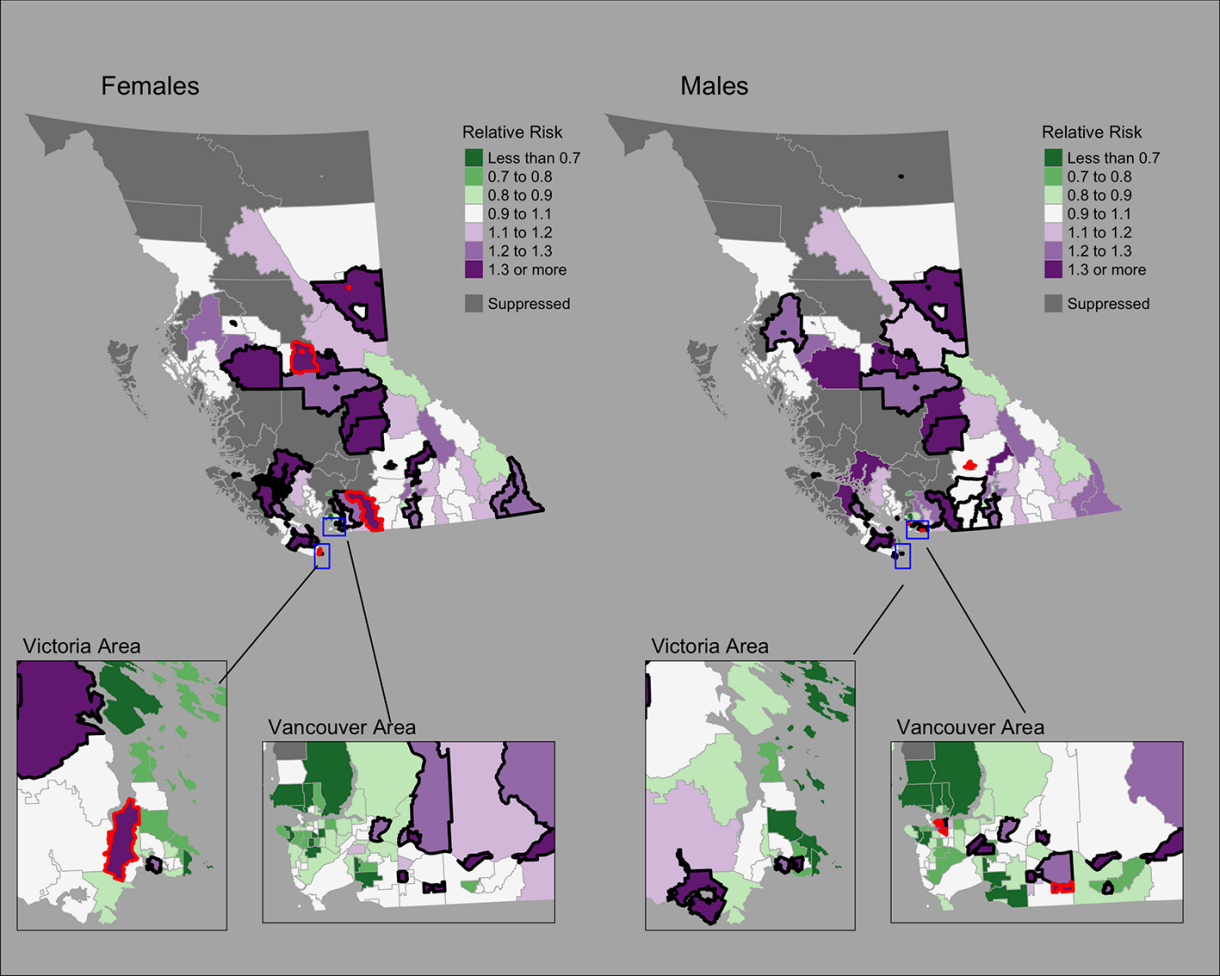
Lung cancer posterior median relative risks by CHSA and sex, 2011–2018. Areas with an exceedance probability of 80% or greater (threshold RR = 1.1) are shown in black bolded borders. Areas shown in red bolded borders correspond to those listed in **Table 1**.

**Figure 1** shows the mapping of posterior median RRs. The five largest RRs among areas at elevated risk are presented in **Table 1**. The spatially structured effect (i.e. the proportion of variance in the modeled RRs explained by spatial structured correlations) was 12.2% (95% CrI = 0.6%–33.8%) and 5.5% (95% CrI = 0.1%–19.9%) among women and men, respectively. Overall, there were N = 50/218 and N = 44/218 CHSAs with elevated risk among women and men, respectively. Among these areas, there were 1245 and 1112 additional cases than expected among women and men, respectively.

**Table 1:** Community Health Service Areas with the largest posterior median relative risks by cancer type, 2011–2018.

Cancer	Sex	CHSA	O	E	SIR	RR	95% CrI	Exceedance probability
Lung	Women	Chetwynd	13	4	3.40	1.67	1.06–2.50	96%
		Vanderhoof Rural	12	3	3.63	1.63	1.02–2.48	95%
		Prince George City – Central	173	106	1.64	1.58	1.36–1.82	100%
		Hope		30	1.75	1.55	1.19–1.96	99%
		Langford/Highlands	129	80	1.61	1.54	1.30–1.81	100%
	Men	Downtown Eastside	174	65	2.69	2.49	2.14–2.88	100%
		Aldergrove/Otter	52	26	1.99	1.71	1.31–2.20	100%
		Prince George City – Central	155	97	1.60	1.54	1.31–1.79	100%
		Cedar Cottage	66	39	1.68	1.53	1.21–1.91	100%
		Kamloops Centre North	202	129	1.57	1.53	1.33–1.75	100%
								
Breast	Women	South Cowichan	167	119	1.41	1.21	1.07–1.38	94%
		Pitt Meadows	135	106	1.27	1.18	1.03–1.35	84%
Cervix	Women	–	–	–	–	–	–	–
Colorectal	Women	City of Langley	101	70	1.45	1.22	1.04–1.43	89%
		Prince George City – Central	125	89	1.40	1.22	1.05–1.42	91%
		Vernon Centre/Coldstream	211	166	1.27	1.17	1.04–1.32	86%
	Men	Haney	114	85	1.34	1.18	1.02–1.37	82%

CHSA, Community Health Service area; O, Observed; E, Expected; SIR, Standardized Incidence Ratio; RR, Relative Risk; CrI, Credible Interval. Expected counts were rounded to the nearest whole value.

Observed and fitted values were generally similar for moderate SIR values but showed deviations near extreme values (**Supplementary Figure 1A**). The PIT plots show a generally uniform pattern with some deviations in the tails (**Supplementary Figure 1B**).

### Female breast cancer

**Figure 2:**
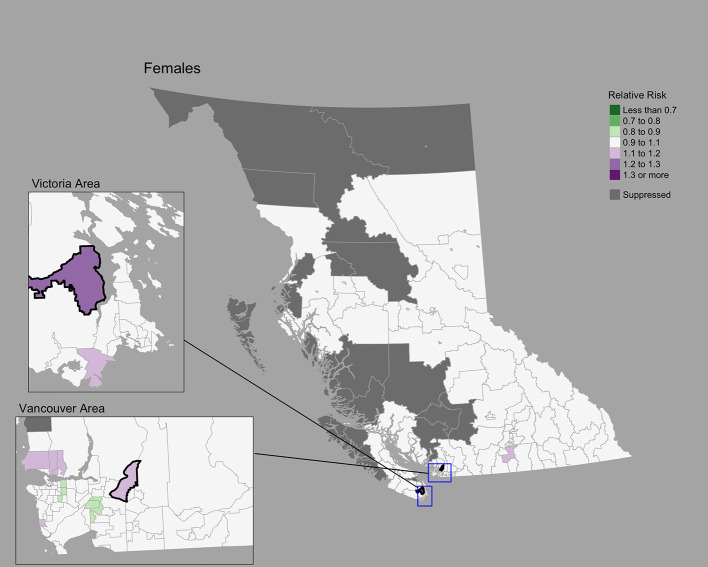
Female breast cancer posterior median relative risks by CHSA, 2011–2018. Areas with an exceedance probability of 80% or greater (threshold RR = 1.1) are shown in black bolded borders.

The Moran’s *I* statistic for female breast cancer SIRs was -0.119 (p = 0.992), not supporting clustering in the data. Female breast cancer cases by CHSA ranged from 0 to 534. Among areas with non-zero counts, SIRs ranged from 0.34 to 3.45. RRs ranged from 0.85 to 1.21. Choropleth maps of RRs are presented in **Figure 2** and areas with elevated risk were presented in **Table 1**. The spatially structured effect was 19.3%. (95% CrI=1.2%-50.7%) Overall, there were two CHSAs with elevated risk, representing 77 excess cases.

Posterior predictive checks are presented in **Supplementary Figures 2A, B**. Observed and fitted values showed large deviations at extreme SIR values; RR’s generally hovered around 1.0 (**Supplementary Figure 2A**). The PIT plots show a generally uniform pattern with deviations at the tail ends (**Supplementary Figure 2B**).

### Cervix cancer

**Figure 3:**
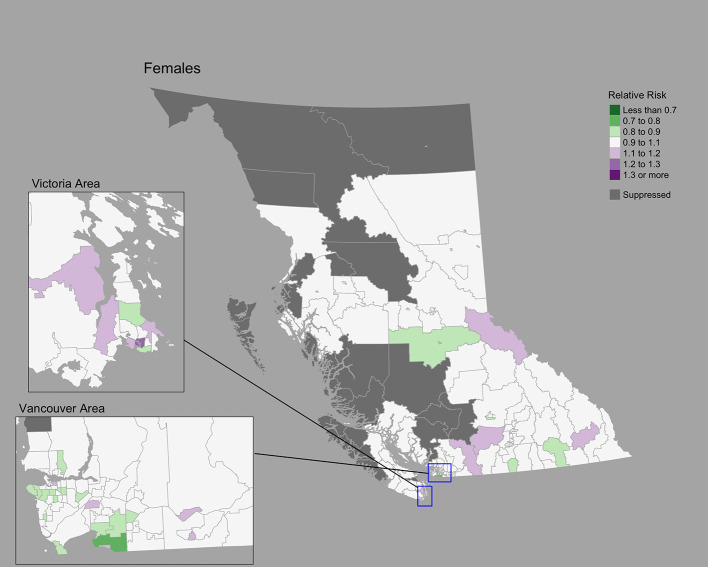
Cervix cancer posterior median relative risks by CHSA, 2011–2018. Areas with an exceedance probability of 80% or greater (threshold RR = 1.1) are shown in black bolded borders.

The Moran’s *I* statistic for cervix cancer SIRs was 0.211 (p = 0.001), indicating significant clustering. Cervix cancer cases by CHSA ranged from 0 to 25. Among areas with non-zero counts, SIRs ranged from 0.17 to 6.4. RRs ranged from 0.80 to 1.27. A choropleth map of RRs is **Figure 3**. Overall, there were no CHSAs with elevated risk. The posterior median spatially structured effect was 7.8% (95% CrI=0%-44.3%).

Posterior predictive checks are presented in **Supplementary Figures 3A, B**. Observed and fitted values showed large deviations for extreme SIRs; RR’s generally remained near 1.0 (**Supplementary Figure 3A**). The PIT plots show a generally uniform pattern (**Supplementary Figure 3B**).

### Colorectal cancer

**Figure 4:**
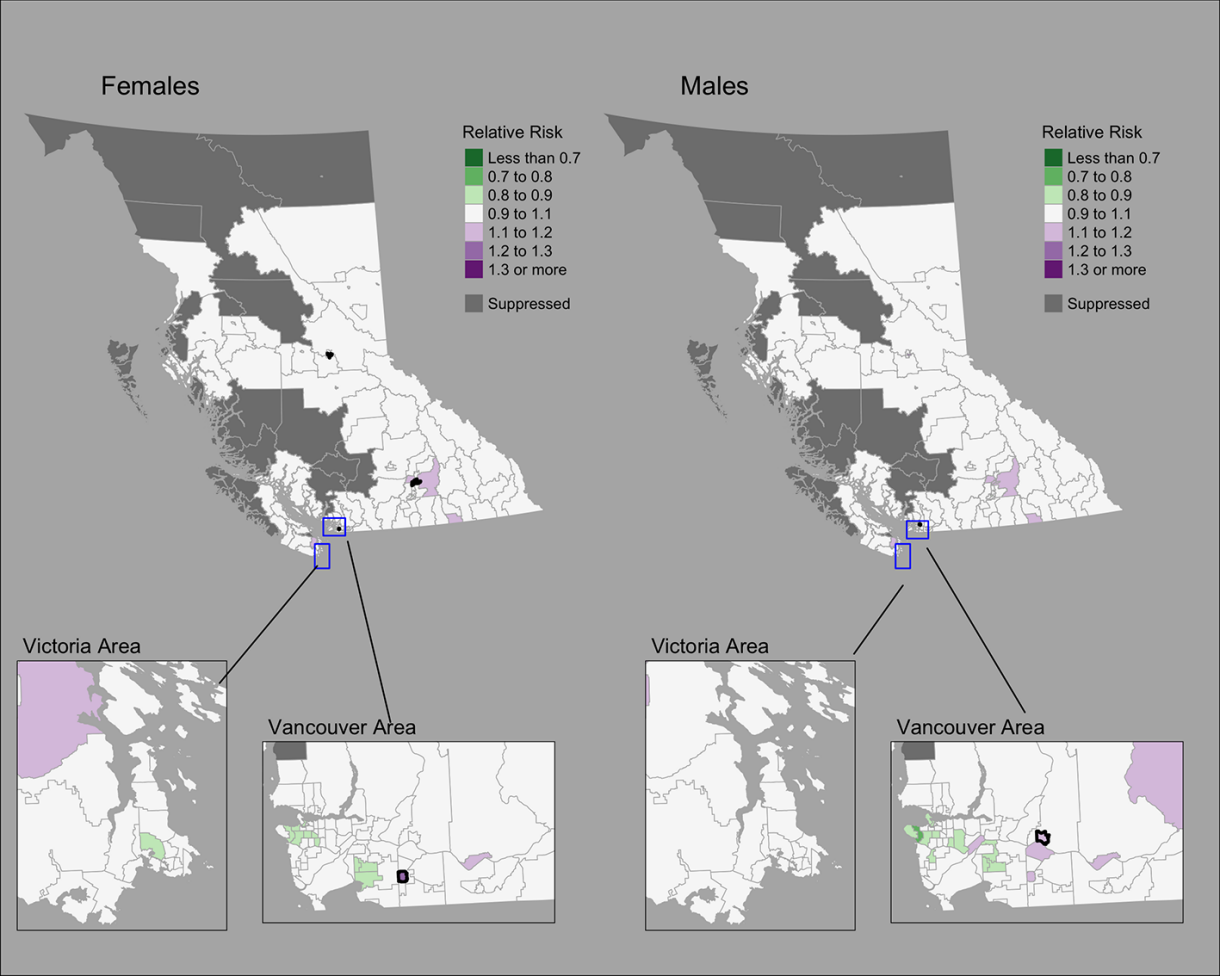
Colorectal cancer posterior median relative risks by CHSA and sex, 2011–2018. Areas with an exceedance probability of 80% or greater (threshold RR = 1.1) are shown in black bolded borders.

The Moran’s *I* statistic among female colorectal cancer SIRs was 0.017 (p = 0.28) and does not support clustering in the data. Among men, the statistic was 0.139 (p = 0.006), indicating significant clustering. Among women, observed colorectal cancer cases by CHSA ranged from 0 to 211 cases. Women’s SIRs ranged from 0.38 to 5.14 among non-zero count areas. Women’s RRs ranged from 0.82 to 1.22. Among men, observed cases by CHSA ranged from 0 to 237 cases. Men’s SIRs ranged from 0.36 to 4.52 among non-zero count areas. Men’s RRs ranged from 0.76 to 1.18. Choropleth maps of RRs are presented in **Figure 4**. CHSAs with elevated risk are presented in **Table 1**. Overall, there were three and one CHSAs with elevated risk among women and men, respectively. Among these regions, there were 111 more female cases than expected and 29 more male cases than expected. The spatially structured effect was 4.6% (95% CrI = 0%-29.0%) and 4.4% (95% CrI = 0%–24.8%) among women and men, respectively.

Observed and fitted values showed large deviations for extreme SIRs (**Supplementary Figure 4A**). The PIT plots show a generally uniform pattern with deviations at the tail ends (**Supplementary Figure 4B**).

## Discussion

### Summary of findings

This study presents results from a technically accessible approach for examining cancer risk at small geographic areas using the R package *smallareamapp* that can be applied to support cancer surveillance, health planning, and health research. This approach includes Bayesian hierarchical models to estimate spatially smoothed RRs, exceedance probabilities, and the spatially structured effect, as well as options for conducting posterior predictive checks and Global Moran’s *I* test for spatial autocorrelation. Using cancer registry data for BC, we present the cancer risk for small geographic areas relative to provincial rates for lung, female breast, colorectal, and cervical cancers. At the CHSA level, which comprises 218 regions across BC, the proportion of variance in the modeled RRs explained by a spatial effect ranged from 4.4% (male colorectal) to 19.2% (female breast), that is, some percentage of the modeled risk have an underlying spatial structure beyond randomness. Lung, cervix, and male colorectal cancers showed significant spatial clustering. Lung cancer showed the greatest number of regions with elevated risk, representing 2357 excess cases during the period 2011–2018.

### Geographic variations of cancer incidence in BC

Strong geographic patterns were identified for lung cancer, consistent with previous reports in BC that primarily focused on large health regions (44, 45). At a small area scale, we found areas that deviated from previously reported large area trends. Among men, the Downtown Eastside CHSA was significantly elevated despite being located in the Vancouver Coastal Health region, where lung cancer incidence rates are the lowest (44). In BC’s North, where lung cancer incidence rates are the highest (44), a number of regions showed lower risk. Similar differences in lung cancer incidence between broad and local scales have been reported elsewhere in Canada (46). Bayesian hierarchical modeling has enabled more accurate assessment of risk among small areas and more detailed information for regional cancer control and prevention (46). Geographic variations in lung cancer, as well as sex-specific trends, are likely related to regional and historical differences in tobacco smoking rates (47, 48), radon exposure (49), and socioeconomic status (50).

Compared to lung cancer, female breast, colorectal, and cervix cancers showed less pronounced regional differences. The conservative nature of the BYM2 model may impact estimates of spatial variation (10), particularly for cancers with low incidence like cervix. Geographic variations may also be related to variations in screening participation, socioeconomic and sociodemographic characteristics, and risk factors (51, 52). To our knowledge, there are no small area studies of cancer screening in BC. Elsewhere in Canada, significant spatial variations of cancer screening for breast, cervix, and colorectal cancers were reported among small areas (53). Among large health regions in BC, breast cancer screening shows differences by geography (51, 52) and material deprivation (51). Significant variations in cervix cancer incidence in BC have been reported by rural-urban classifications, ethnicity/race, smoking, and marital status (54).

Compared to other provinces, age-standardized incidence rates (ASIR) for various cancer types are generally lower in BC (all-cancer ASIR, both sexes, 501.8 per 100,000) compared to the rest of Canada (all-cancer ASIR, both sexes, 556.3 per 100,000), which includes 13 provinces and territories (48). Among the four cancer types examined in this paper, BC has the lowest male and female lung cancer ASIR (Men: 55.2 per 100,000; Women: 54.3 per 100,00) in Canada (48). In respect to colorectal cancer, the BC male ASIR (61.1 per 100,000) is the second lowest in Canada and the BC female ASIR (46.7 per 100,00) is on par with the Canadian ASIR (48). Female breast cancer ASIR (116.4 per 100,000) is the third lowest in Canada and the cervix ASIR (6.5 per 100,000) is the second lowest in Canada (48). Geographic differences in cancer incidence across provinces and territories in Canada are attributable to variations in modifiable risk factors, environmental influences, access and availability of cancer services, and the social determinants of health. Diagnostic practices can also influence geographic variation in cancer incidence, as has been shown with geographic variations in prostate-specific antigen testing and prostate cancer incidence.

### Small area risk estimation using BYM2

A common challenge in population oncology has been unstable risk estimates for areas with sparse event data (14, 18, 21). However, recent work has improved estimation and measures of uncertainty for small areas (9, 21, 55, 56). Spatial smoothing through Bayesian hierarchical models is one approach that increases risk estimate stability by borrowing information from neighboring areas (9, 20, 21). Duncan et al., used the Bayesian Leroux model (57) and Markov Chain Monte Carlo methods to examine cancer incidence and survival across 2148 small areas in the Australian Cancer Atlas (9). The BYM model and INLA were used by Moraga in the SpatialEpiApp (20) and by Saint-Jacques for modeling the risk of bladder and kidney cancer (19, 55). Brown et al. calculated the spatial intensity surface of lung and thyroid cancer using a log-Gaussian Cox Process and INLA (56, 58). Even in the absence of spatial autocorrelation, Bayesian hierarchical models are valuable for reducing noise in small area analysis (35).

We used the BYM2 model (29, 31), a re-parameterization of the BYM model proposed by Simpson et al that addresses two issues: (1) the spatially structured and unstructured effect terms are not identifiable, and (2) the spatially structured term is not scaled (29, 31). A simulation study by Riebler et al. found that BYM2 performed at least as well as other models, including BYM, Leroux, and Dean’s model (29). Others have reported preference for BYM2 over BYM to separately identify the spatial and unstructured effects (59) and when using zero-inflated data (60).

Our approach builds off prior examples of small area risk estimation in population oncology. Richardson et al. reported that BYM was a conservative approach to risk estimation (10). A simulation study found high specificity even when data were sparse, but sensitivity was low when the elevated risk was moderate (RR <2.0) or based on expected counts less than 50 (10). Others have proposed using the whole posterior distribution to improve sensitivity (10) and interpretation (61). Richardson et al. recommended exceedance probabilities of 70–80% (10). These recommendations were applied to various neighborhood and community level analyses of cancer incidence (19, 35, 55). Holowaty et al. suggested using Bayesian smoothing together with other spatial statistics such as Moran’s *I* to assess global clustering, which we implemented (35). As suggested by Holowaty et al., we used Moran’s *I* as an initial assessment of the entire study area for clustering in the data (35).

### As an accessible analytic approach and tool for cancer surveillance and research

Dawe et al. recommended that Bayesian spatial modeling be more widely used to inform the planning of cancer screening and prevention strategies (46). Despite advances in methodologic approaches that enable small area risk estimation, technical capacity and expertise to carry out analyses vary regionally (14, 18, 35). In both Canada and the United States, the majority of geospatial studies in population oncology were concentrated among a few cancer registries (14) and NCI-designated Cancer Centers (18), respectively. Holowaty et al. suggested data access and analytical capacity as important limitations for many public health units (35). To address existing gaps in analytic capacity, we packaged the methods presented in this study into the *smallareamapp* R package (22) for public use. The package can be implemented as an R Shiny (62) app, which can be easily used by epidemiologists and analysts for small area risk estimation and data visualization. Our app builds off of similar functionality in the SpatialEpiApp (20), a Shiny app and R package for general spatial and spatio-temporal analysis (20). Our package enables the use of BYM2 and INLA following the approach and purposes described in this study and contains posterior predictive checks and formal tests for spatial autocorrelation.

While we do not incorporate covariate risk factor data, such information can be readily added to the BYM2 model and assessed in light of spatial dependencies. Morales-Otero et al. applied BYM2 to study infant mortality in Colombia and examined associations with sociodemographic factors (59). Morris et al. used BYM2 to model school-age pedestrian injuries from motor vehicle crashes in New York City and explored associations with sociodemographic factors (63). In Canada, socioeconomic and demographic Census data are available at the neighborhood level through the Canadian Index of Multiple Deprivation (64) and Material and Social Deprivation Index (65). The *smallareamapp* R package, however, does not currently have the ability to include fixed effects. This functionality will be considered for future iterations. Model extensions to account for temporal correlations and spatio-temporal interactions are also possible (32), as demonstrated by Moraga in the SpatialEpiApp (20) with the Bernardinelli model (66).

### Strengths

The present study examines 8 years of data obtained from the BCCR, a population-based cancer registry gold-certified by the North American Association Central Cancer Registries. Nearly 98% of tumor records were geocoded. To our knowledge, this is the first small area study of cancer risk in BC in peer-reviewed literature. We used the CHSA unit, which is the smallest health administrative area in BC. Compared to traditional non-spatial analyses in cancer surveillance, the present study makes full use of available health data in a BYM2 model framework (29, 31). Analyses conducted at larger administrative levels (e.g. the 89 Local Health Areas versus 218 CHSAs) typically lack sufficient resolution to be used for localized surveillance and planning. We also implemented models using INLA, which provides a rapid and robust estimation within a Bayesian framework without the need for time-consuming MCMC sampling. The *smallareamapp* R package is freely available and open source, and may be readily used by others for investigating spatial variation in risk (22).

### Limitations

Despite CHSAs being the smallest health administrative area level in BC, more work is required to determine the optimal geographic scale and spatial weighting for modeling cancer risk (67). In this study, a complete six-digit postal code was assigned to a geocoordinate using the Statistics Canada PCCF+ program. The PCCF+ uses population weighting and random allocation to inform the geocoordinate of postal codes, specifically when postal codes match to multiple potential reference points (a geocoordinate that links to a small Census geographical unit, such as a blockface or a dissemination area) (26). Positional accuracy is better among urban versus rural areas. However, the magnitude of positional error (distance error between PCCF+ geocoordinates and true reference point) is typically small and more serious for highly localized exposure assessment. The positional error in this study is not expected to impact the assignment of CHSA to cases from a complete six-digit postal code because the CHSA is a relatively large area compared to postal codes and the magnitude of PCCF+ positional errors reported in prior literature (26, 68, 69).

While this study focused on identifying areas with an elevated risk, this approach can be also used to examine areas of low incidence. This may be relevant in the context of cancer screening to evaluate prevention and early detection.

Sensitivity analyses to assess the effect of different hyperprior specifications on posterior risk estimates and the spatial effect were not conducted in this study. The primary purpose of this study was to demonstrate the utility of the *smallareamapp* R package, and in doing so, we followed specifications chosen by Riebler et al. (29). The ability to add custom hyperprior specifications for the PC prior, as well as alternate prior choices, are not yet available in the *smallareamapp* R package but will be considered for future updates. In this study, the spatial effect appeared low in some cases, despite a relatively strong Moran’s *I* value (e.g. male lung cancer). This observation is not clear and to our knowledge, the relationship between the Moran’s *I* statistic and the modeled spatial effect has not been explored. Further work is required to interpret the spatial effect in relation to Moran’s *I*.

The modeled relative risks and model’s spatial effect are influenced by the spatial weights matrix, which is user-defined. A queen’s contiguity weights matrix was chosen. While these weights are often considered a standard choice in many spatial analytical tools, this approach does not account for islands in spatial autocorrelation because islands do not share any common borders or vertices (i.e. they have zero neighbors). In this study, 3 of 218 areas were considered islands. The modeled relative risks among these areas were not adjusted for spatial effects, as they do not have neighbors, but they are still adjusted for unstructured random effects. Compared to the SIR values, the modeled RRs for these areas generally moved closer towards the null risk (RR = 1.0). Another option to consider when islands are present is the k-nearest neighbor weights (KNN). The KNN weights consider the first k-neighbors, irrespective of their distance to a given area. These are not yet available in the *smallareamapp* R package but can be introduced and will be considered for future updates.

Even though risk can be estimated for areas with sparse data (including zero counts), Bayesian disease-mapping models like BYM and BYM2 are considered conservative (10). Following recommendations by various studies (10, 19, 35, 55, 61), we leveraged information from the whole posterior distribution through exceedance probabilities to improve sensitivity in detecting areas with a high probability of having an elevated risk, although posterior predictive performance highlighted some model deficiencies, in particular for cervical cancer where case counts were relatively small. Bayesian hierarchical models are only one approach to disease mapping and risk estimation. While we implemented Bayesian smoothing together with Moran’s *I*, other methods could be considered, such as the spatial scan statistic (35).

### Conclusions

Small area analyses in cancer surveillance are increasingly warranted; however, analytic capacity remains limited. In this study, we draw on methodological advances to present a technically accessible approach and tool for small area risk estimation and disease mapping using the BYM2 model. Our *smallareamapp* R package can be used by epidemiologists and surveillance analysts, as well as health planners, to make full use of georeferenced health data for examining geographic variation in cancer risk. These methods can also be extended to generate hypotheses and examine ecological associations while adjusting for spatial dependencies.

## Data availability statement

The dataset used and analyzed during the current study were obtained from the BC Cancer Registry and are not publicly available due to privacy legislation and institutional data sharing agreements. Data however can be requested through a data access request to BC Cancer following their processes at http://www.bccancer.bc.ca/health-professionals/professional-resources/bc-cancer-registry/request-registry-data. The R package presented in this paper and source code is publicly available on GitHub: https://github.com/jdsimkin04/smallareamapp. A technical walkthrough of the R package available on GitHub and as a supplementary file. Requests to access these datasets should be directed to datareq@bccancer.bc.ca.

## Ethics statement

The studies involving human participants were reviewed and approved by the University of British Columbia - Children’s and Women’s Hospital Research Ethics Board (H19-01100). Written informed consent from the participants’ legal guardian/next of kin was not required to participate in this study in accordance with the national legislation and the institutional requirements. Written informed consent was not obtained from the individual(s) for the publication of any potentially identifiable images or data included in this article.

## Author contributions

All authors contributed to the data acquisition and study design. JS analyzed the data and led the development of the smallareamapp R package. All authors provided feedback to the R package functionality. All authors interpreted the data. JS wrote the first draft of the manuscript and all authors contributed to manuscript revisions. All authors read and approved the final manuscript.

## Funding

This study was supported by the Canadian Institutes of Health Research through the Canada Graduates Scholarships Doctoral Award. The scholarship provides financial support for students in doctoral studies. JS is a recipient of this award. The Canadian Institutes of Health Research has no involvement in development of this manuscript.

## Conflict of interest

The authors declare that the research was conducted in the absence of any commercial or financial relationships that could be construed as a potential conflict of interest.

## Publisher’s note

All claims expressed in this article are solely those of the authors and do not necessarily represent those of their affiliated organizations, or those of the publisher, the editors and the reviewers. Any product that may be evaluated in this article, or claim that may be made by its manufacturer, is not guaranteed or endorsed by the publisher.

## Supplementary material

Supplementary Figure 1 Posterior predictive checks: **(A)** scatterplot of observed lung cancer SIRs and fitted values (RR); **(B)** PIT value distribution.

Supplementary Figure 2 Posterior predictive checks: **(A)** scatterplot of observed female breast cancer SIRs and fitted values (RR) and **(B)** PIT value distribution.

Supplementary Figure 3 Posterior predictive checks: **(A)** scatterplot of observed cervical cancer SIRs and fitted values (RR) and **(B)** PIT value distribution.

Supplementary Figure 4 Posterior predictive checks through a **(A)** scatterplot of observed colorectal cancer SIRs and fitted values (RR) and **(B)** PIT value distribution.

Supplementary Figure 5 Posterior predictive checks through a histogram of PIT values.

Supplementary Figure 6 Posterior predictive checks through histogram and density plots of Pearson residuals.

Supplementary Figure 7 Posterior predictive checks through observed and fitted counts.

## Abbreviations

BC, British Columbia; BCCR, British Columbia Cancer Registry; BYM, Besag, York and Mollie; BYM2, Modified Besag, York and Mollie; CHSA, Community Health Service Area; CrI, Credible Intervals; E, Expected; HA, Health Authority; INLA, Integrated Nested Laplace Approximation; KM, kilometer; O, Observed; PC, Penalized Complexity; PIT, Predictive Integral Transformed; RR, Relative Risk; SIR. Standardized Incidence Ratio.
