# Supplementary figures and images for: Small area disease mapping of cancer incidence in British Columbia using Bayesian spatial models and the smallareamapp R Package
Source: Front Oncol. 2022 Oct 19;12:833265. doi: 10.3389/fonc.2022.833265 (PMC9627310; doi:10.3389/fonc.2022.833265)

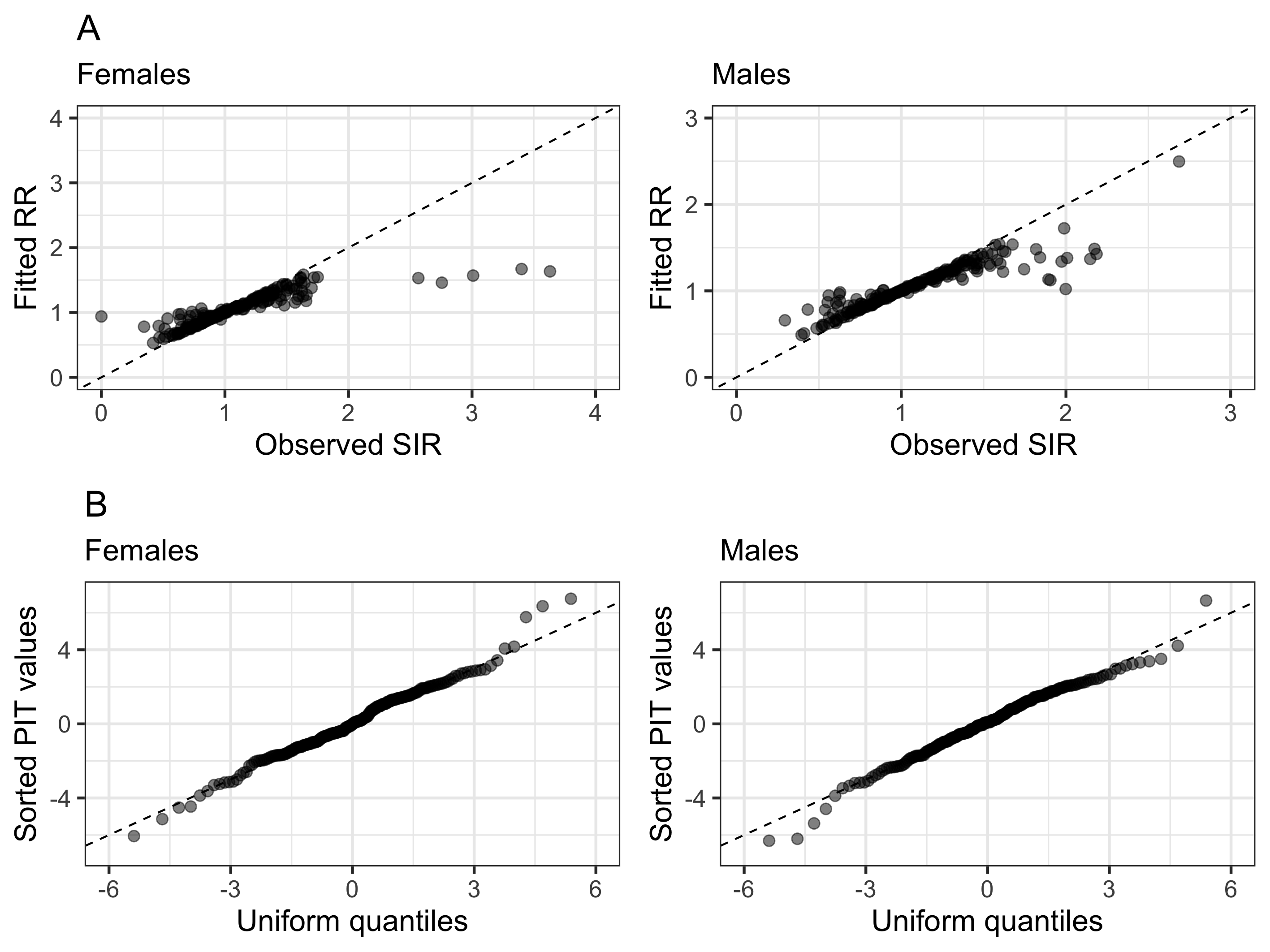

Supplement: Supplementary Figure 1 — Posterior predictive checks: (A) scatterplot of observed lung cancer SIRs and fitted values (RR); (B) PIT value distribution. [file Image_1.png]

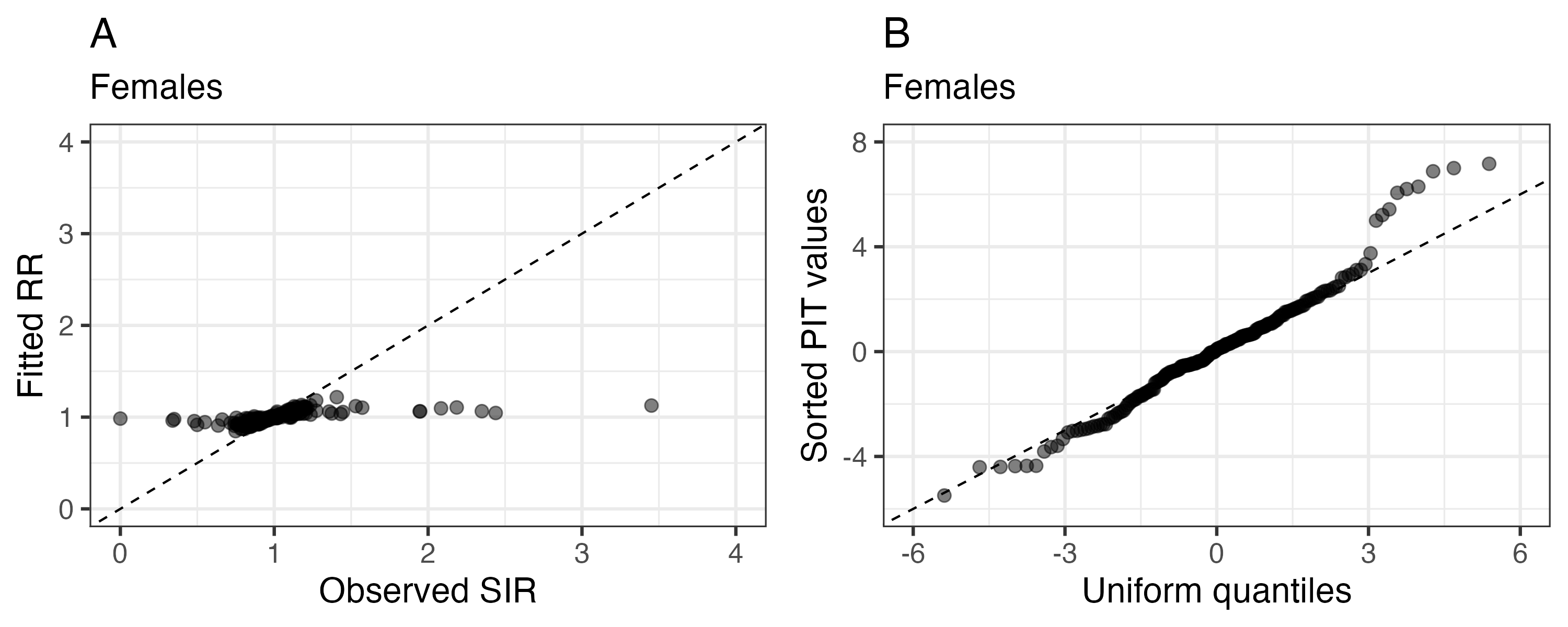

Supplement: Supplementary Figure 2 — Posterior predictive checks: (A) scatterplot of observed female breast cancer SIRs and fitted values (RR) and (B) PIT value distribution. [file Image_2.png]

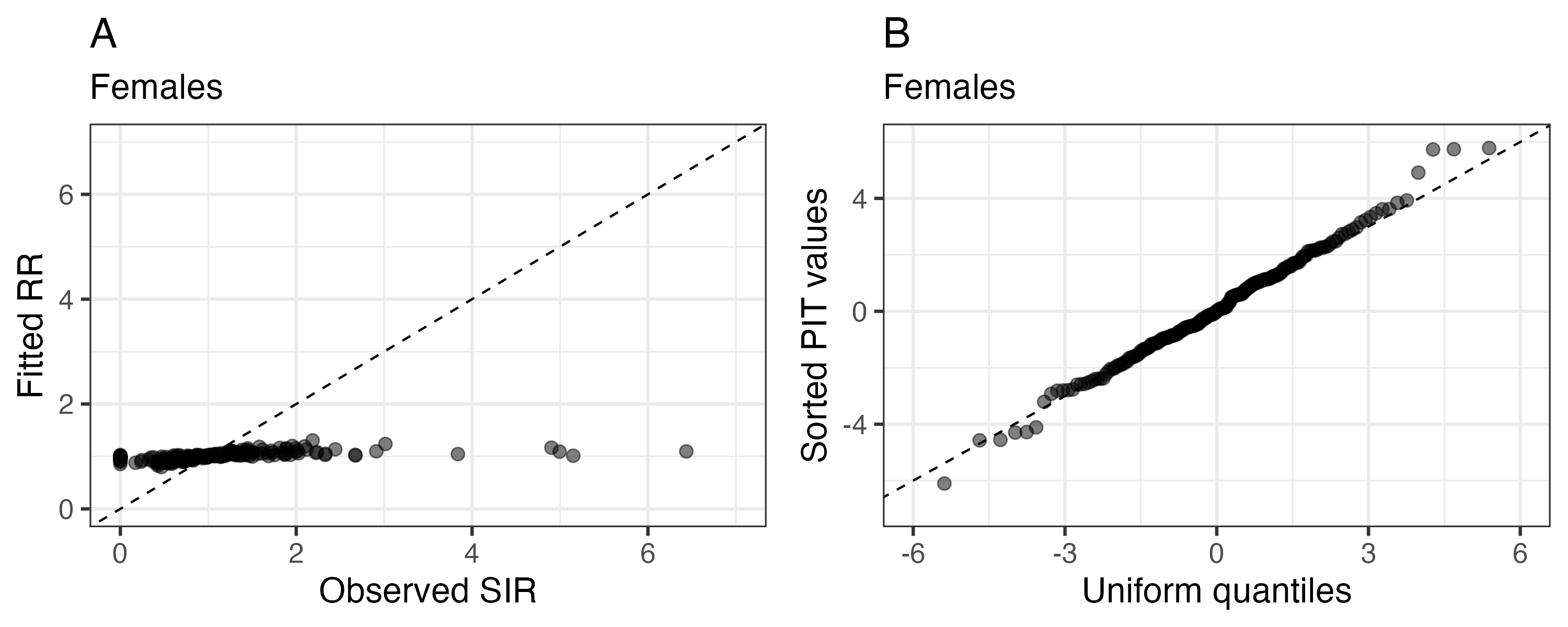

Supplement: Supplementary Figure 3 — Posterior predictive checks: (A) scatterplot of observed cervical cancer SIRs and fitted values (RR) and (B) PIT value distribution. [file Image_3.png]

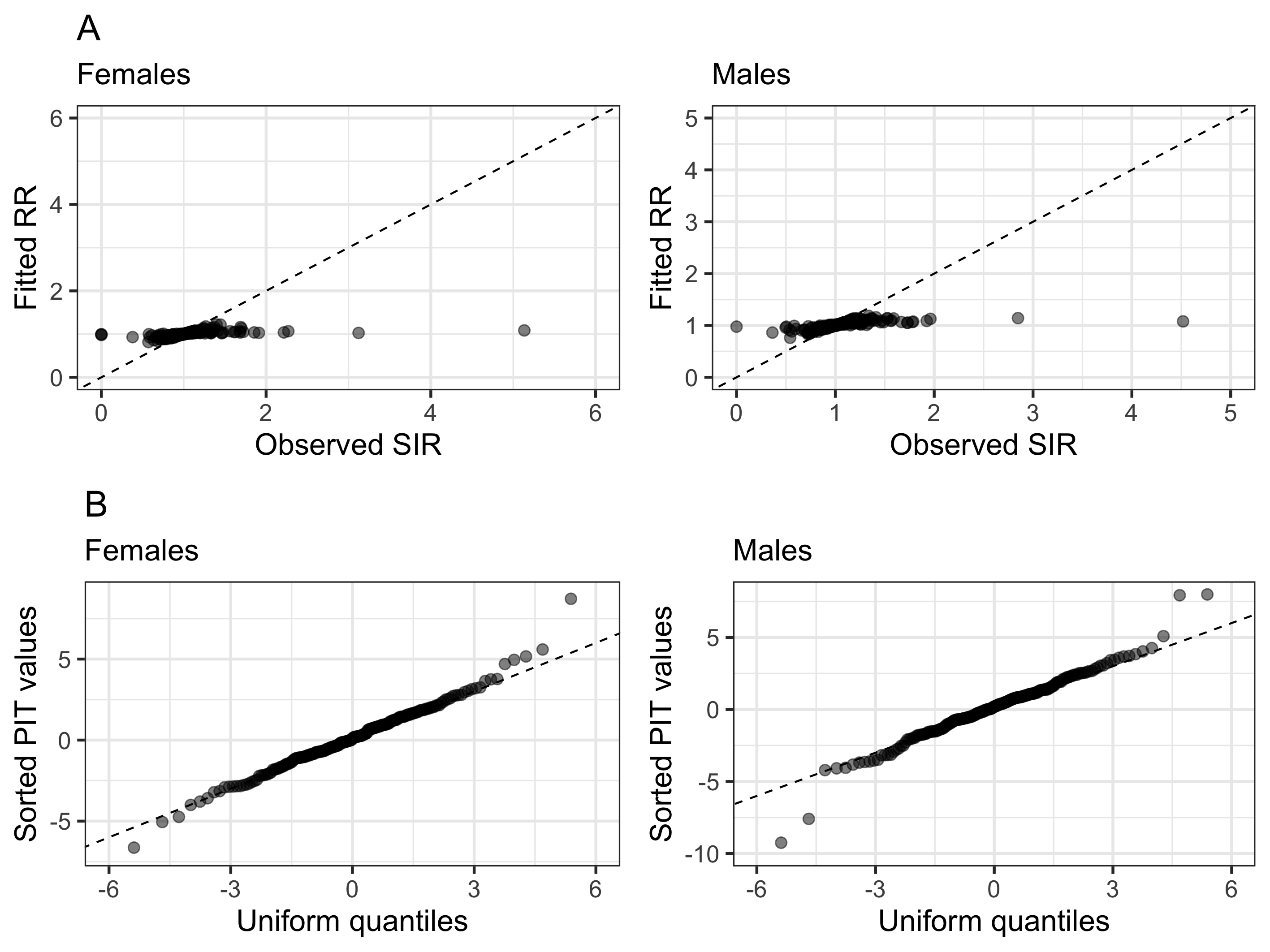

Supplement: Supplementary Figure 4 — Posterior predictive checks through a (A) scatterplot of observed colorectal cancer SIRs and fitted values (RR) and (B) PIT value distribution. [file Image_4.png]

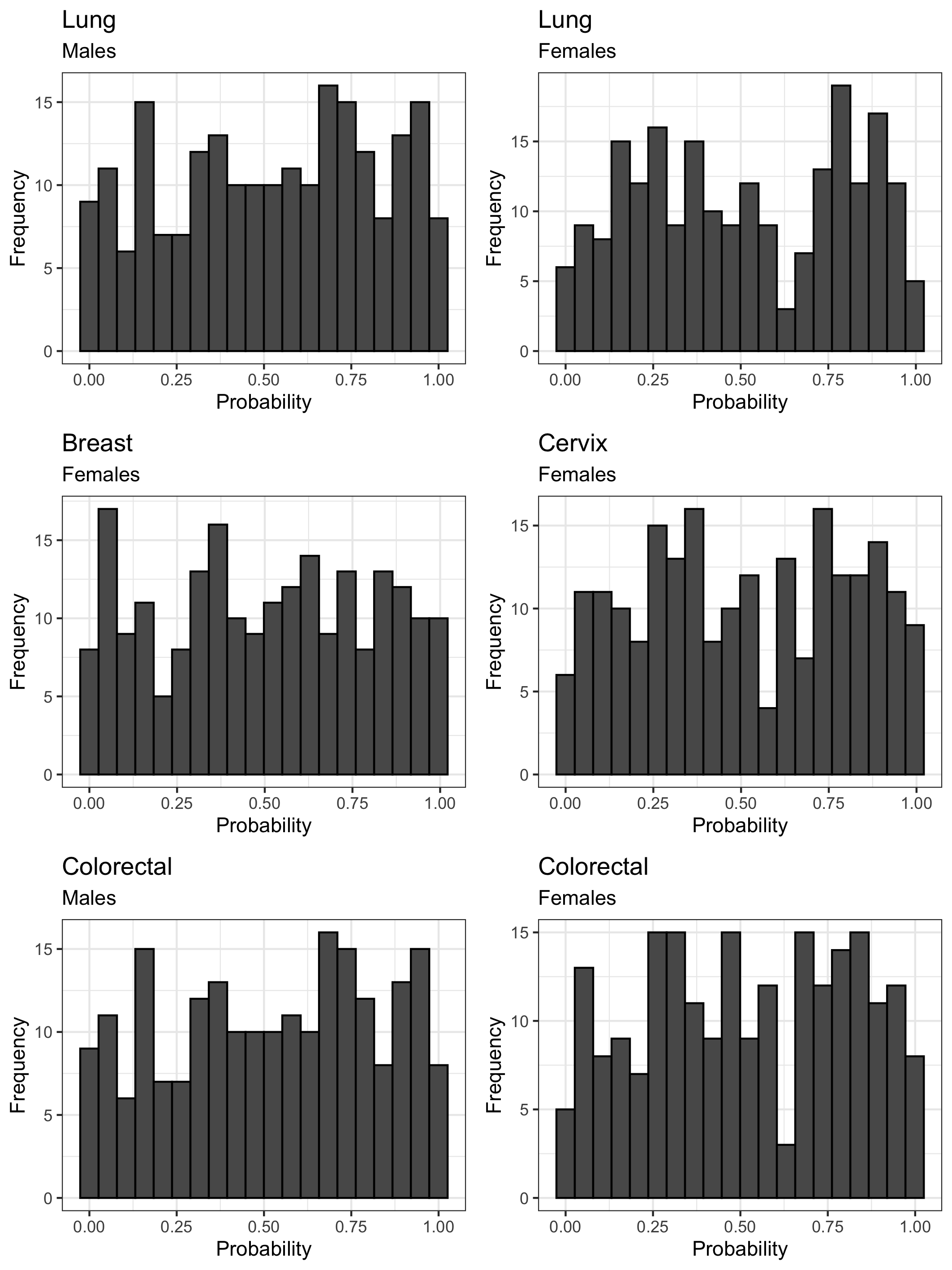

Supplement: Supplementary Figure 5 — Posterior predictive checks through a histogram of PIT values. [file Image_5.png]

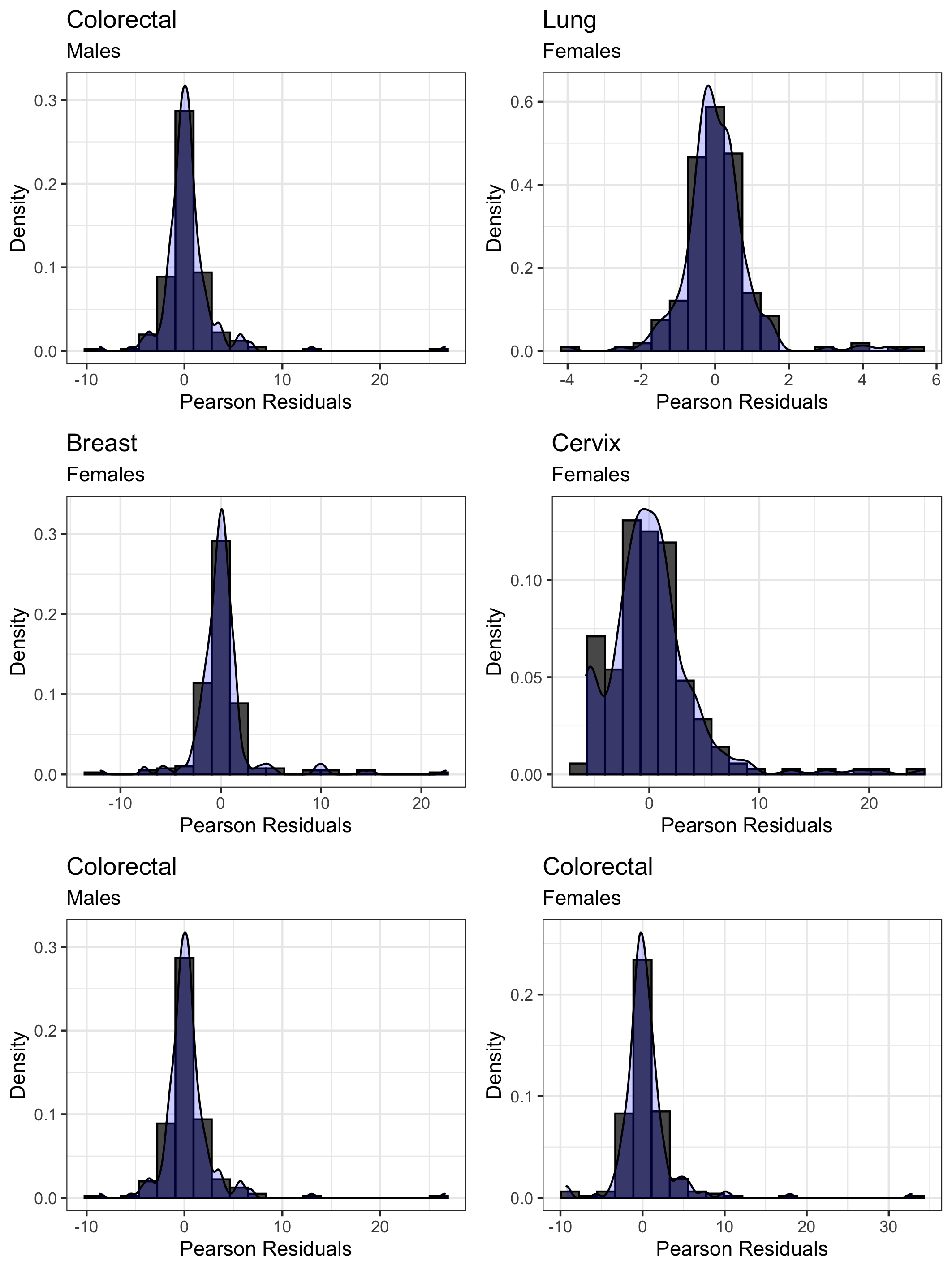

Supplement: Supplementary Figure 6 — Posterior predictive checks through histogram and density plots of Pearson residuals. [file Image_6.png]

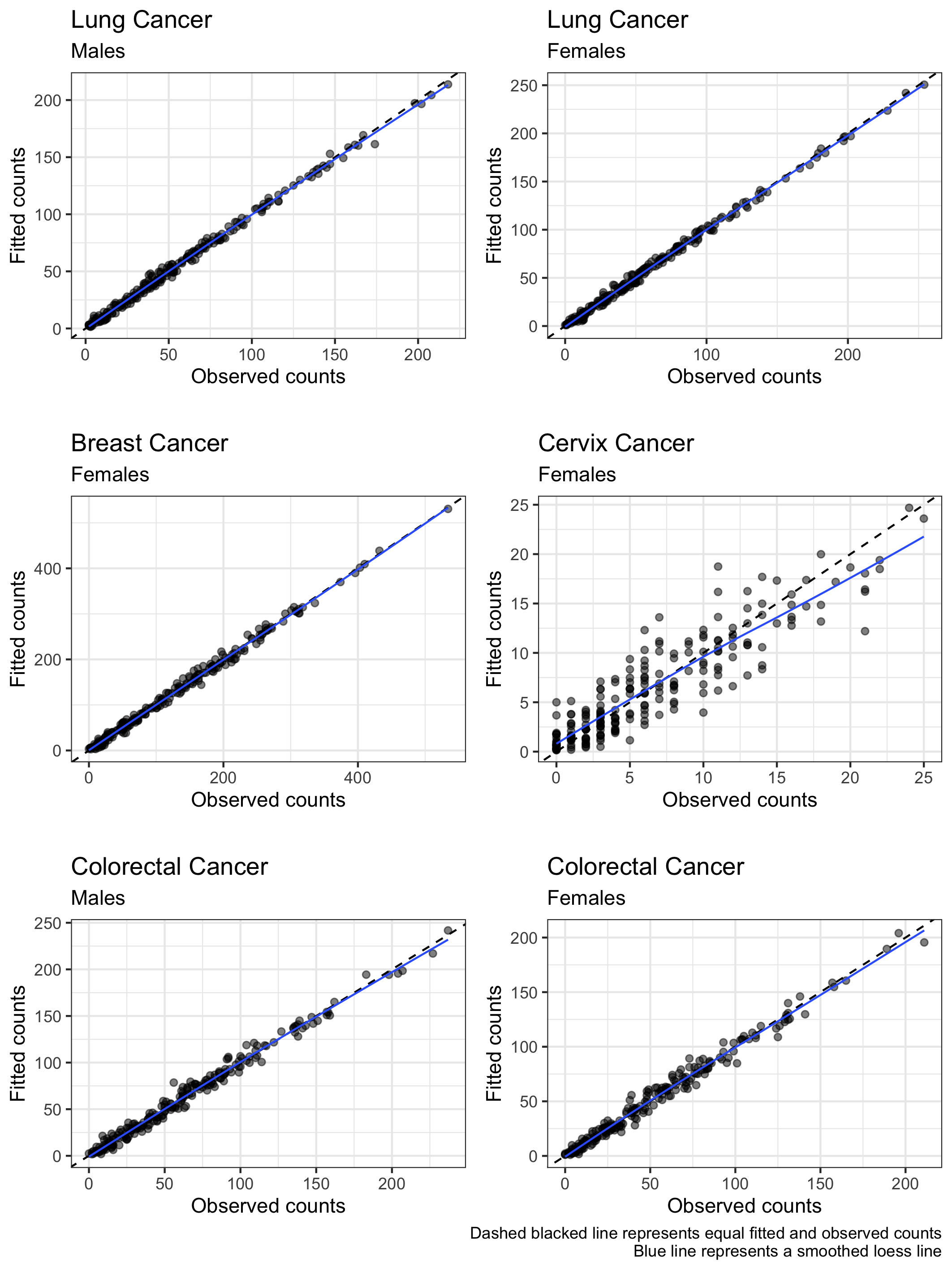

Supplement: Supplementary Figure 7 — Posterior predictive checks through observed and fitted counts. [file Image_7.png]
