# Supplementary material for: Small area disease mapping of cancer incidence in British Columbia using Bayesian spatial models and the smallareamapp R Package
Source: Front Oncol. 2022 Oct 19;12:833265. doi: 10.3389/fonc.2022.833265 (PMC9627310; doi:10.3389/fonc.2022.833265)
Supplement: Supplementary file 8 [file DataSheet_1.docx]

#### APPENDIX A

#### The Besag,York and Mollie model (BYM) and the modified BYM (BYM2)

The BYM model^1^ is a common and long used model that produces smoothed risk estimates by borrowing information from neighbouring areas. The BYM model is a well-established approach and commonly used in epidemiology and hence, provides results that can be compared with other studies. BYM is also readily implementable in available software and a highly feasible approach.

The BYM model includes a spatial random effect (si) that is used to smooth data according to a neighbourhood structure (e.g. a spatial weights matrix defined by Queen’s Contiguity)^1^. The BYM model also includes an unstructured random effect (vi) that is used to model uncorrelated variation^1,2,3^. The BYM model is an extension of what is considered the Intrinsic Conditional Autoregressive (ICAR) model^1,2,3^. One particular issue with the ICAR model is it assumes complete spatial correlation, which is a restrictive assumption and not realistic^1,2^. To address this limitation, the BYM extends the ICAR with an additional parameter (the unstructured random effect)^2,4^. The prior assigned to the spatial random effect is the ICAR^2,4^. The unstructured random effect is considered an independent and identically distributed (iid) normal variable with zero mean and variance^2,4^. The fourth stage includes hyperpriors on the precision hyperparameters of the priors assigned in stage 3^2,4^. In the R-INLA program, the log gamma hyperprior is assigned as a default.

Stage 1: $Yi \sim Poisson\left( Ei\theta i \right)$ for i = 1,..., N areas

Stage 2: $\log(\theta)= intercept+si+ \upsilon i$

Stage 3: ICAR and IID with zero mean and variance

Stage 4: log gamma (0.1, 0.1)

There are two limitations to the BYM model and how it is constructed, which has led to various derivatives of the BYM model, including Dean and Leroux^4^. The first limitation is that the structured and unstructured random effects are not identifiable^4^. The second limitation is that the precision hyperparameters of the random effects are not scaled and therefore, do not represent variability on the same level^4^. While the Leroux model and Dean model propose ways to address the first limitation, they do not address the second^4^. Riebler *et al.* discuss this in detail and present the BYM2 as a solution to both limitations^4^. Briefly, the BYM2 is a modification of the Dean model^4^, and ultimately, a re-parameterization of the random effects in BYM as follows:

Random effect = $\frac{1}{\sqrt{\tau}}(\sqrt{1-\phi}v+ \sqrt{\phi}s)$

In this case, *s* (scaled spatial effect) and *v* (unstructured effect) are standardized to have a variance equal to one^4,5^. The BYM2 introduces two new parameters $\tau$(the marginal precision) and $\phi$ the mixing parameter^4,5^. The marginal precision parameter controls the marginal variance contribution by the *s* and *v*^4,5^. The mixing parameter distributes the variance between *s* and *v*^4,5^. Essentially, the mixing parameter represents the fraction of marginal variance explained by the spatial term^4,5^. There are hyperpriors assigned to $\frac{1}{\sqrt{\tau}}$ and $\phi$, which are referred to as penalized complexity (PC) priors, which are described by Simpson *et al.* and Riebler *et al.* in more detail^4,5^.

**References**

1. Besag J, York J, Mollie A. Bayesian image restoration, with two applications in spatial statistics. Ann Inst Stat Math. 1991;43(1):1–20.
2. Duncan EW, Cramb SM, Baade PD, Mengersen KL, Saunders T, Aitken. JF. Developing a Cancer Atlas using Bayesian Methods: A Practical Guide for Application and Interpretation. [Internet]. Brisbane; 2019. Available from: https://atlas.cancer.org.au/developing-a-cancer-atlas/index.html#suggested-citation
3. 424. Moraga P. Geospatial Health Data: Modeling and Visualization with R-INLA and Shiny [Internet]. Chapman & Hall; CRC Biostatistics Series; 2019 [cited 2021 Jan 2]. Available from: https://www.paulamoraga.com/book-geospatial/
4. Riebler A, Sørbye SH, Simpson D, Rue H. An intuitive Bayesian spatial model for disease mapping that accounts for scaling. Stat Methods Med Res. 2016;25(4):1145–65.
5. Simpson D, Rue H, Riebler A, Martins TG, Sørbye SH. Penalising Model Component Complexity: A Principled, Practical Approach to Constructing Priors. Stat Sci [Internet]. 2017;32(1):1–28. Available from: http://dx.doi.org/10.1214/16-STS576.http://hdl.handle.net/10754/623413
