# Supplementary material for: Small area disease mapping of cancer incidence in British Columbia using Bayesian spatial models and the smallareamapp R Package
Source: Front Oncol. 2022 Oct 19;12:833265. doi: 10.3389/fonc.2022.833265 (PMC9627310; doi:10.3389/fonc.2022.833265)
Supplement: Supplementary file 9 [file Presentation_1.pdf]

# APPENDIX B: GETTING STARTED WITH THE *SMALLAREAMAPP* R PACKAGE

## OVERVIEW

*smallareamapp* is an R package for small area risk estimation through Bayesian hierarchical models and disease mapping. Development of *smallareamapp* was motivated by the need for technically accessible approaches to small area risk estimation in population oncology. It is intended for epidemiologists and surveillance analysts to make full use of georeferenced health data for examining geographic variation in cancer risk.

The package takes advantage of R-INLA, an R package for Bayesian model implementation through Integrated Nested Laplace Approximation (INLA)[1]. INLA is an alternative methodology to MCMC methods, and provides a robust and rapid method for Bayesian implementation. The *smallareamapp* primarily runs through an interactive Shiny application (or 'app'). R Shiny is a package and framework that enables development of interactive apps featuring data visualization and statistical computation.

# Table of Contents

**Overview** ........ 1

**System requirements** ........ 3

*Dependencies* ........ 3

**Download and installation** ........ 3

**Using smallareamapp** ........ 4

*Getting started* ........ 4

*App structure* ........ 4

*Welcome page* ........ 5

*File Upload page* ........ 5

**A** File upload if you don't have data and are using the example dataset ........ 5

**B.** Instructions if you are uploading your own disease event data ........ 7

*Analytics page* ........ 8

Analytics sidebar menu ........ 9

*Getting started with spatial model tab* ........ 10

The Map card ........ 11

Datatable card ........ 12

Selecting "Yes" to Implement Spatial modelling w/ INLA button ........ 12

Excess cases card ........ 13

Spatial structured effect card ........ 14

*Model validation subpage* ........ 14

*Spatial autocorrelation subpage* ........ 15

**References** ........ 16

## SYSTEM REQUIREMENTS

The *smallareamapp* R package was written in R, a free and open source programming language. The tool is intended to be used with R versions 4.0.0 (April 2020) or later and RStudio version 1.2.1335 or later. Earlier versions of R will not necessarily support this tool and must be updated prior to installation. Please note that methods carried out in *smallareamapp* are technically sophisticated and require sufficient RAM.

The *smallareamapp* relies on various R packages to run. Users must have read/write access to a local or network folder for installing and updating R packages. Firstly, users are required install R-INLA. The stable version of R-INLA can be installed with the following R code in your R console:

```
install.packages("INLA",repos=c(getOption("repos"),INLA="https://inla.r-inla-download.org/R/stable"), dep=TRUE)
```

Please refer to the R-INLA website for more details on download and installation.

### DEPENDENCIES

Dependencies beyond R-INLA are shown below. When installing *smallareamapp* for the first time, R may prompt you to install these dependency packages. Installing these packages for the first time may take a while.

- R version >= 2.10, shiny, readr, magrittr, dplyr, DT, viridis, viridisLite, kableExtra, sf, tmap, tmaptools, spdep, shinycssloaders, shinyscreenshot, bs4Dash, shinydashboardPlus, INLA, gtools, plotly

## DOWNLOAD AND INSTALLATION

The *smallareamapp* can be installed from it's GitHub repository with the following code.

```
devtools::install_github("jdsimkin04/smallareamapp")
```

Either the remotes or devtools R packages will be required to download *smallaeramapp* from the Github repository. This may take a significant amount of time, please ensure enough downtime to install these packages.

Before using the *smallareamapp* on your own data, we encourage you to familiarize yourself with the app's functionality. The next few sections provides a tutorial for using the *smallareamapp* package.

# USING SMALLAREAMAPP

## GETTING STARTED

1. Load the package into your R environment:

```
library(smallareamapp)
```

*smallareamapp* is designed to run a Shiny app locally that allows you to upload, model and visualize disease event data with a point-and-click approach. Currently, *smallareamapp* has one core function, runmApp(), which launches the Shiny app.

2. Launch the app with the following code:

```
runmApp()
```

R will launch a Shiny app in a new window and open to the *Welcome* page (Figure 1).

**Figure 1.** Welcome page for the *smallareamApp.*

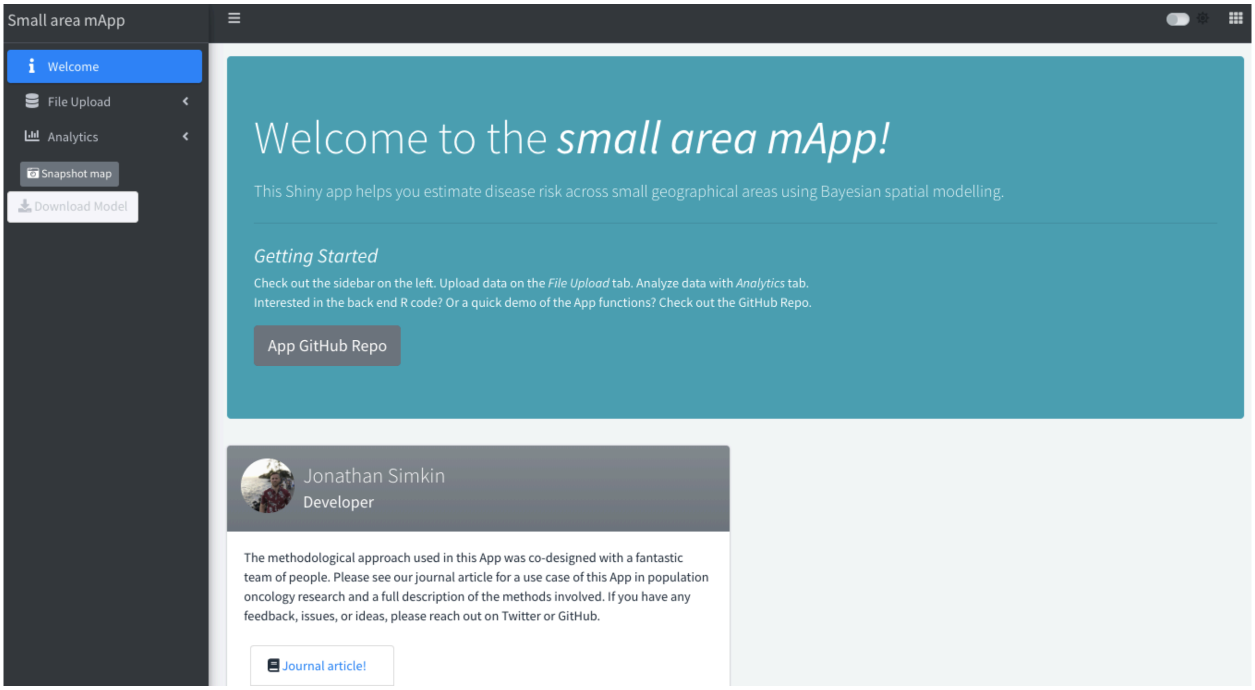


## APP STRUCTURE

The app is organized into three main pages – *welcome*, *file upload*, and *analytics*. The app design follows the bsdash R package structure.

- There is a **navigation bar** on the left that can be toggled by the three bar indicator on the top left. Use the left navigation bar allows to move through the three App pages.

- The **main panel** with app content is in the center. In the main panel, content is packed into different "boxes" or "cards", that can be collapsed or opened.
- There is an optional **right menu bar** for analytics toggled by the 3 x 3 squares on the top right.

## WELCOME PAGE

The *Welcome* page includes a title, brief description, instructions to get started, and a link to the GitHub repository. A card is shown on near the bottom including information on the App developer, acknowledgement of the scientific team, and external links to relevant publications, twitter, and GitHub.

1. To get started, use the navigation bar on the left and click the *File Upload* page.

## FILE UPLOAD PAGE

This page is divided into two sub-pages – *disease event data* and *geography shapefile* (Figure 2). On these two sub-pages, you can upload your data and map shapefiles, or select an example dataset that is provided within the app. Specifically, the app requires a CSV file with disease event data and a corresponding geography shapefile.

**Figure 2.** Upload disease event data on the File Upload page, Disease event data sub-page.

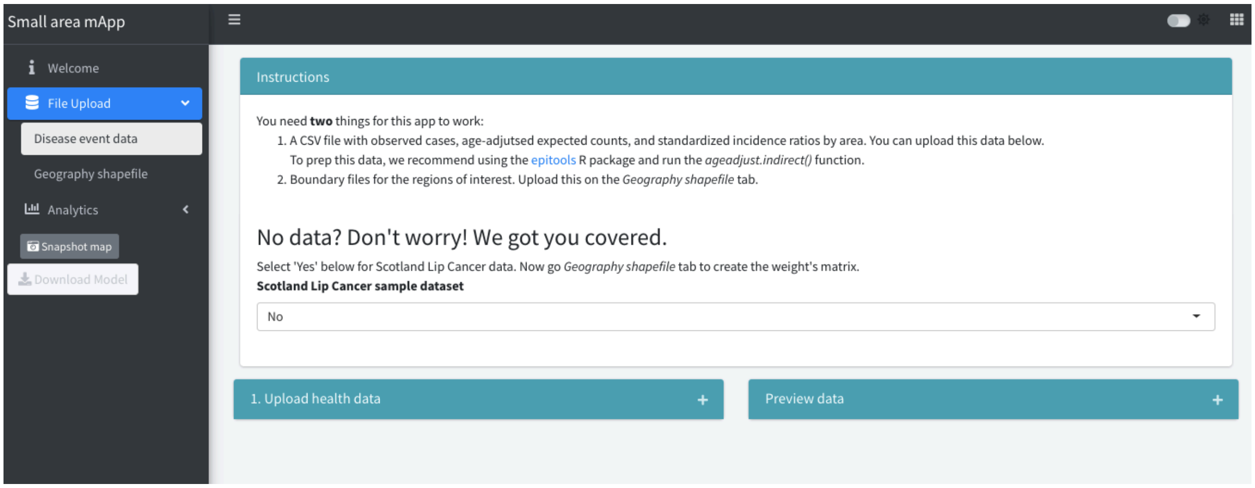


### *A FILE UPLOAD IF YOU DON'T HAVE DATA AND ARE USING THE EXAMPLE DATASET*

1. In the main panel, see the **instructions card**. Select Yes in the drop down menu. This will load the Scotland lip cancer sample dataset[2,3]. Note the Scotland shapefile will uploaded as well.

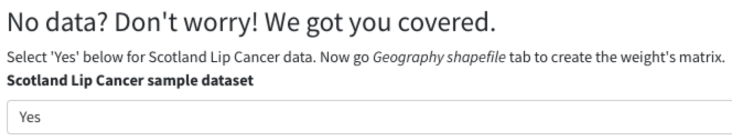


2. You can preview the dataset by expanding the **upload health data card** and click the **update preview table button**. Expand the **Preview data card** by clicking the "+" on the top right of the card (Figure 3).

**Figure 3.** File upload page, example dataset shown in the preview data card.

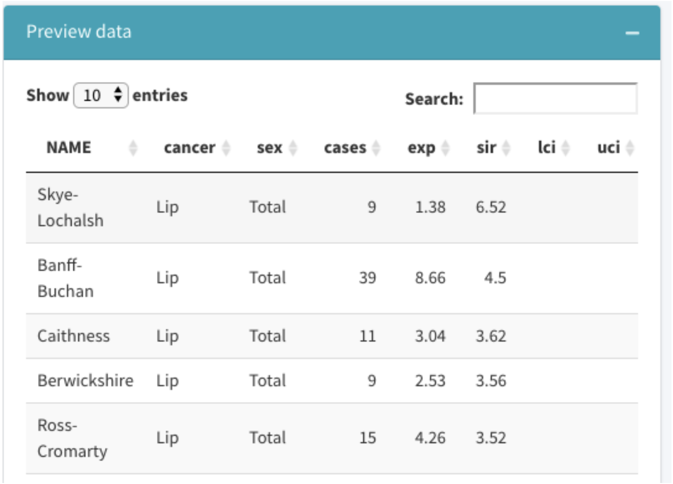


| NAME | cancer | sex | cases | exp | sir | lci | uci |
|---|---|---|---|---|---|---|---|
| Skye-Lochalsh | Lip | Total | 9 | 1.38 | 6.52 | | |
| Banff-Buchan | Lip | Total | 39 | 8.66 | 4.5 | | |
| Caithness | Lip | Total | 11 | 3.04 | 3.62 | | |
| Berwickshire | Lip | Total | 9 | 2.53 | 3.56 | | |
| Ross-Cromarty | Lip | Total | 15 | 4.26 | 3.52 | | |

3. Click on the *Geography shapefile* sub-page in the left navigation bar, under the *File Upload* page tab.
4. The Scotland map will already be uploaded. Don't upload any shapefiles.
5. If you want to preview the map, expand the **Preview Map card** by clicking the "+" button on the top right (Figure 4).

**Figure 4.** File upload page, example dataset shapefile in the preview map card.

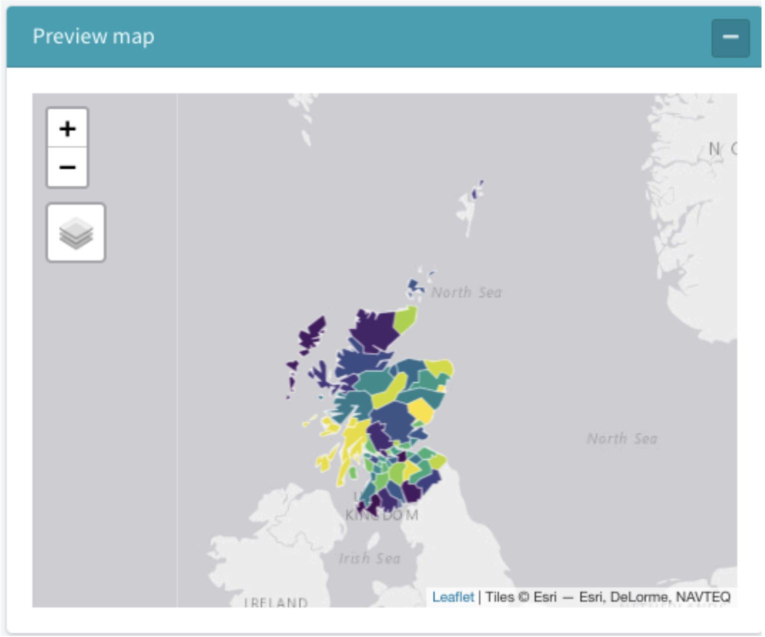


6. To carry out small area risk estimation, you must create a spatial matrix. Click the **Create Weights Matrix button**. A text message below the button will indicate when the weights are generated (Figure 5).

**Figure 5.** File upload page, creating a weights matrix for small area risk estimation.

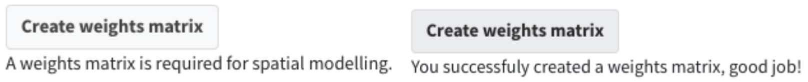


### *B. INSTRUCTIONS IF YOU ARE UPLOADING YOUR OWN DISEASE EVENT DATA*

1. In the main panel, see the **instructions card** and follow the instructions.
2. For step 1 (as specified in the card), expand the **Upload health data card** with the "+" button on the top right. Upload a CSV file by clicking the **browse button** (Appendix Figure 6).
   - **NOTE:** At this time, data must be entered with the specific variables, order and column names as described in the card. A template dataset can be seen by expanding the **Preview data card**, or by clicking on **Reset to template table button** and viewing in the **Preview data card**. This also means that data must be prepped in advance and formatted as noted above. This includes observed counts, age-adjusted expected counts, standardized incidence ratios and 95% confidence intervals, by area, cancer type and sex. Users are expected to know how to calculate standardized incidence ratios. This can be done in R with the epitools R package using the ageadjust.indirect() function.

**Figure 6.** File upload page, uploading health data card.

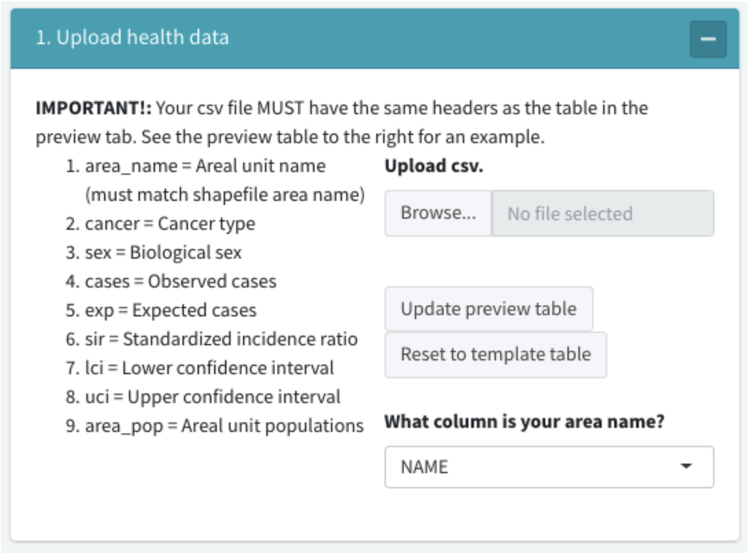


3. After uploading health data, you can preview it by clicking on the **preview table button** on the **upload health data card** and expanding the **preview data card** (Figure 7)

**Figure 7.** File upload page, previewing template dataset.

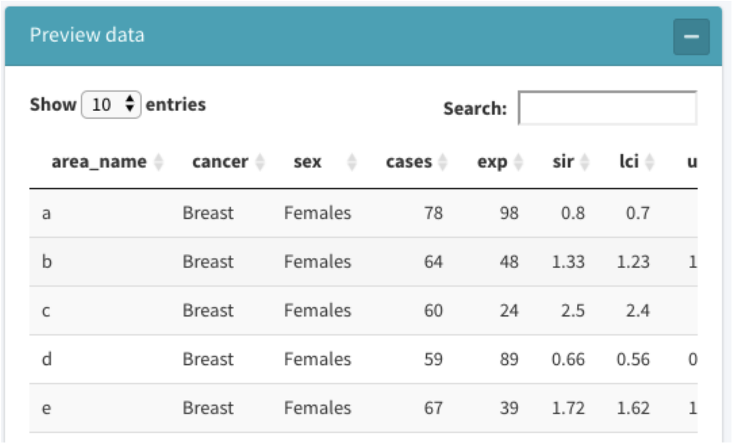

Preview data

Show 10 entries Search:

| area_name | cancer | sex | cases | exp | sir | lci | u |
|---|---|---|---|---|---|---|---|
| a | Breast | Females | 78 | 98 | 0.8 | 0.7 | |
| b | Breast | Females | 64 | 48 | 1.33 | 1.23 | 1 |
| c | Breast | Females | 60 | 24 | 2.5 | 2.4 | |
| d | Breast | Females | 59 | 89 | 0.66 | 0.56 | 0 |
| e | Breast | Females | 67 | 39 | 1.72 | 1.62 | 1 |

4. Go to the *Geography shapefiles* sub-page.
5. Click the **browse button** to upload the corresponding geography shapefiles. This can include multiple files and extensions, typically .shp, .shx, .prj, and .dbf.
    - **NOTE:** Geography shapefiles can often be obtained from government institution websites. Canadian maps can be downloaded at the Statistics Canada website: https://www12.statcan.gc.ca/census-recensement/2011/geo/bound-limit/bound-limit-2016-eng.cfm.
6. Once uploaded, use the dropdown menu to select the shapefile data field that represents the area name. This must be the same as the area name in the CSV file uploaded earlier in step 2.
7. Click on the **Create weights matrix button**. A message will appear under the button once the weights matrix is generated.
    - **NOTE:** This may take up to 30 seconds or longer depending on the complexity of the data. Larger datasets and more connections between areas typically take longer and require more computer memory. A few *benchmarks* are provided here below.

      *System specs: macOS Big Sur; Processor: 1.6 GHz Dual-Core Intel Core i5; Memory: 8GB 1600 MHz DDR3*
        - BC Census Subdividsion areas , N=737, ~11 seconds, file sizes = ~13 MB.
        - BC Community Health Service Areas, N=218, ~ 23 seconds, file sizes = ~14 MB

8. With all your files uploaded and weights matrix created, go to the *Analytics* page and *Spatial Modelling* sub-page to analyze and visualize data.

## Analytics Page

There are three sub-pages in the *Analytics* page – *spatial modeling, model validation,* and *spatial autocorrelation*. The main analytics page is the *spatial modelling* page (Figure 8)

**Figure 8.** Analytics page, Spatial modelling sub-page.

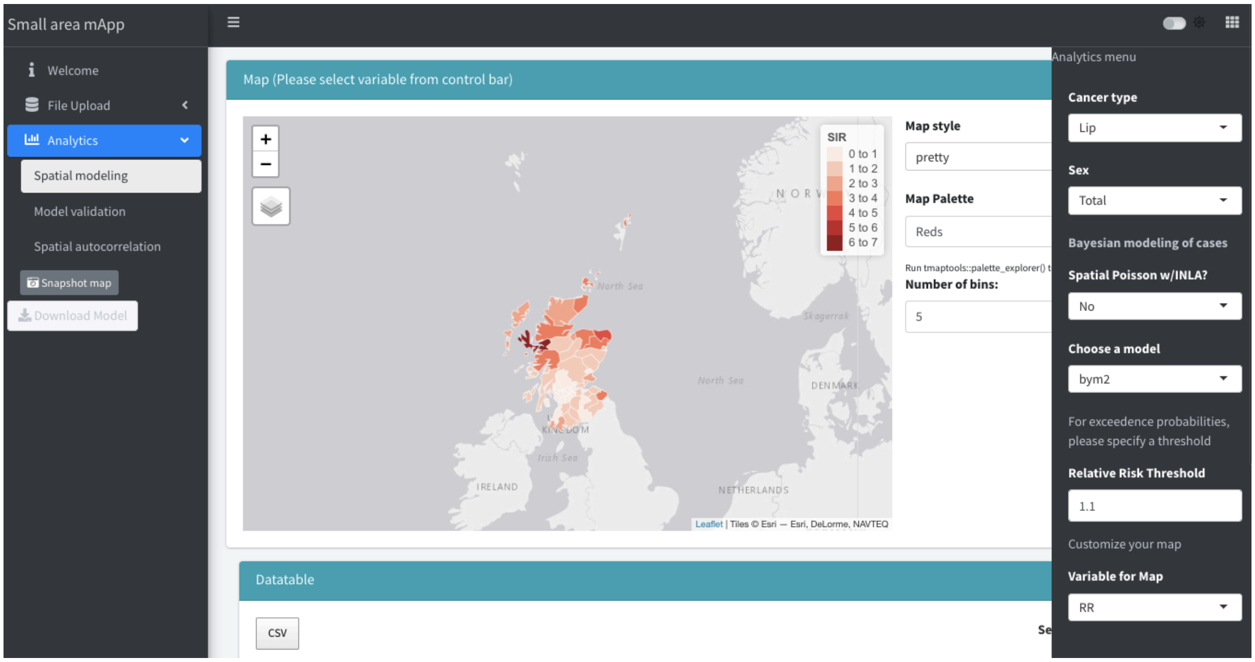


The *Spatial modeling* sub-page includes 4 cards – *map*, *datatable*, *excess cases*, and *spatially structured effect*. A **right side analytics menu sidebar** (Figure 9) will pop up as soon as you enter the page. To hide it or have it re-appear, click the 3x3 squares on the top right of the app.

### *Analytics sidebar menu*

The menu has a series of dropdowns to filter the data, whether to model data and visualize (Figure 9).

**Figure 9.** Analytics sidebar menu.

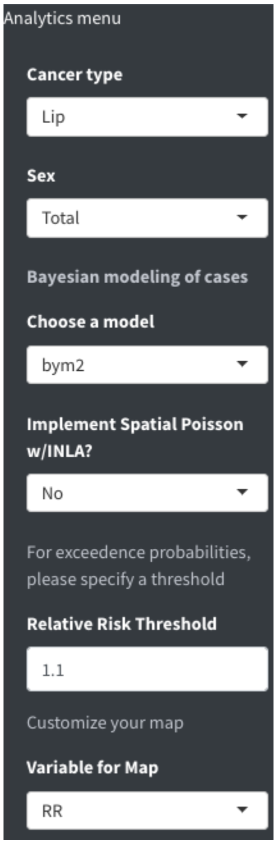


## Menu filters and data options

- *Cancer type*: The unique cancer types included in the uploaded CSV file, specifically in the *cancer* column. Only one can be selected at a time.
- *Sex*: The unique sex categories included in the uploaded CSV file, specifically in the *sex* column. Only one can be selected at a time.
- *Implement Spatial Poisson w/ INLA*?: A Yes/No dropdown selection. The default is "No" and when "No" is selected, all results will remain as they were imported – as the counts observed, expected, standardized incidence ratios, etc. The map card will only show non-modelled values. Selecting "Yes" will initiate a spatial model, either BYM2 or BYM as selected in the following filter *choose a model*.
    - **NOTE:** A spatial model is not required to visualize the SIR and case data.
    - **NOTE:** Initiating the BYM or BYM2 model may take a few minutes to run.
- *Relative Risk Threshold*: Exceedance probabilities are calculated in relation to a relative risk threshold. The default is a threshold of 1.1 or 10% increased risk. Enter a new value to change the threshold.
- *Variable for Map*: Select the variable that you wish to visualize in the map in the main panel. The default is the SIR when the spatial modelling is set to "No" and the RR when it is set to "Yes".

### GETTING STARTED WITH SPATIAL MODEL TAB

1. Use the **analytics sidebar menu** filters and data options to select your inputs of interest. Do not select

"Yes" to the Spatial Modelling at this time. First we will explore the contents on this page.

### *THE MAP CARD*

The **map card** is shown in the main panel area (Figure 10).

**Figure 10.** Map card in the spatial modelling subpage.

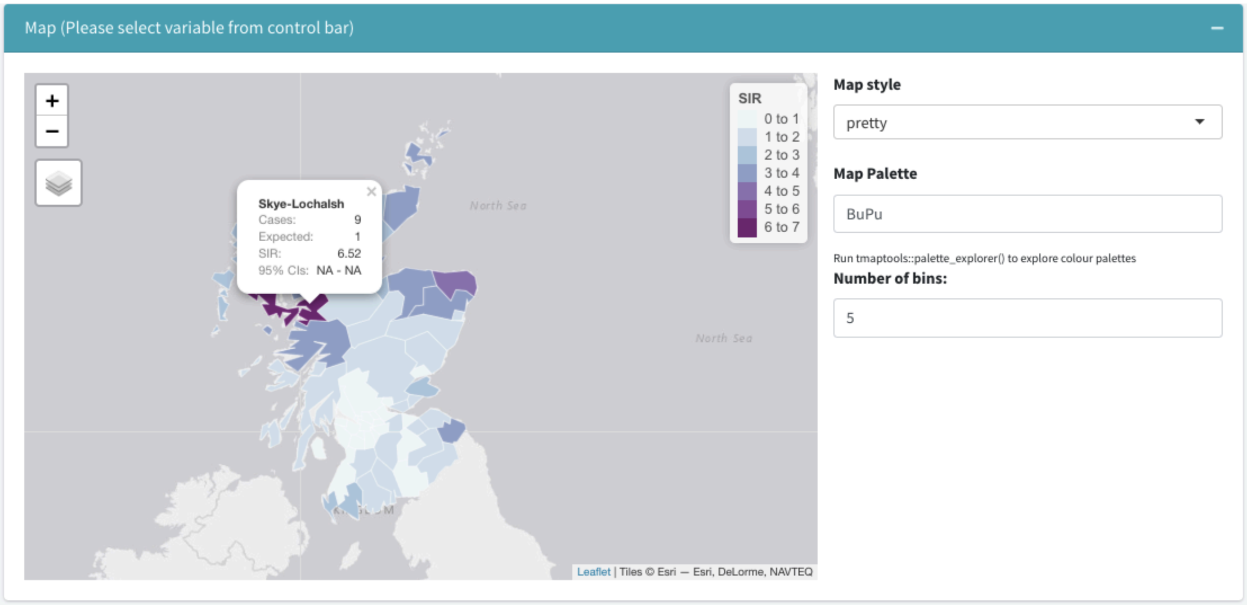


- The SIRs will be mapped by default. You can choose what variable to map in the **analytics sidebar menu** with the **Variable for Map button**.
- The map is interactive leveraging the tmap R package. Click on an area to toggle a pop up window that highlights area level data. You can move around the map with your mouse and zoom in and out of the map.
- Customize the map with the dropdowns on the right side of the card – *map style, map palette,* and *number of bins*.
  - *Map style:* Dropdown with four map styles to choose from, see here for more details on map styles.
  - *Map palette:* This is a free text bar will only accept valid palettes. The default is "BuPu" palette (i.e. blue and purple). We recommend running the tmaptools package palette_explorer() function to explore palettes. Try out "Reds"!
  - *Number of bins*: Select a numerical value to indicate the number of categories for the map. Note that the number of bins does not always correspond with the map produced by tmap.
- Save the map as a .png with the **snapshot button** on the **left navigation bar** (Figure 11).

**Figure 11.** Snapshot button to capture a png image of the map.

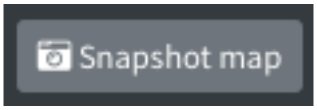


### *Datatable card*

The *datatable card* is shown in the main panel area under the *map card* (Figure 12).

**Figure 12.** The datatable card.

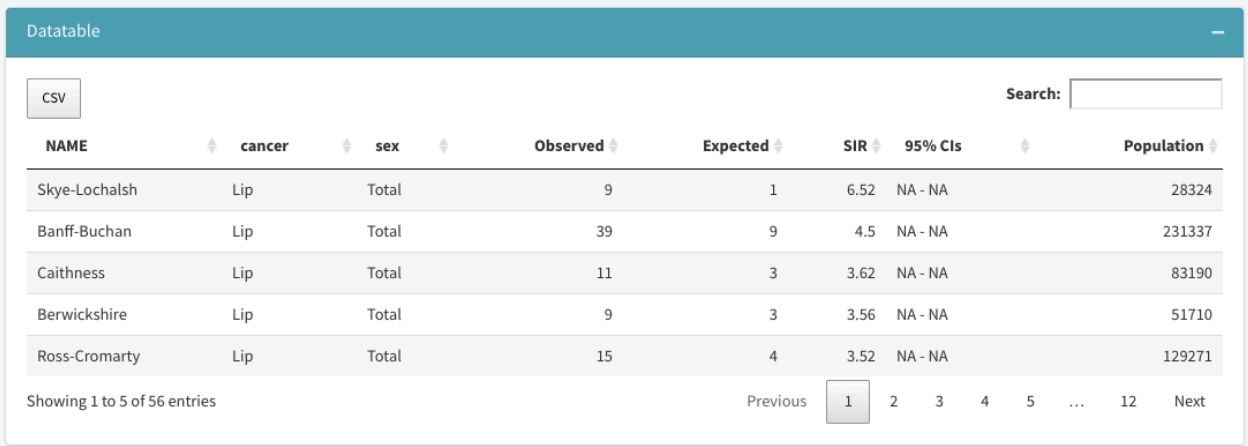

Datatable

CSV

Search:

| NAME | cancer | sex | Observed | Expected | SIR | 95% CIs | Population |
|---|---|---|---|---|---|---|---|
| Skye-Lochalsh | Lip | Total | 9 | 1 | 6.52 | NA - NA | 28324 |
| Banff-Buchan | Lip | Total | 39 | 9 | 4.5 | NA - NA | 231337 |
| Caithness | Lip | Total | 11 | 3 | 3.62 | NA - NA | 83190 |
| Berwickshire | Lip | Total | 9 | 3 | 3.56 | NA - NA | 51710 |
| Ross-Cromarty | Lip | Total | 15 | 4 | 3.52 | NA - NA | 129271 |

Showing 1 to 5 of 56 entries

Previous 1 2 3 4 5 … 12 Next

- The datatable is generated using the package DT. The table is interactive. You can filter the table for terms or numbers with the search bar on the top right and sort the columns by clicking the headers.
- The table will by default show 5 rows. Scan more rows by selecting the “Next” button on the bottom right or selecting one of the numerical pages.
- Download the entire table using the **CSV button**.

### *Selecting “Yes” to Implement Spatial modelling w/ INLA button*

1. In the **analytics sidebar menu**, select the “BYM2” model from the **Choose a model dropdown**
2. To implement the Bayesian model using INLA, select “Yes” to the **Implement Spatial Model dropdown**.
    - **NOTE**: Implementing the BYM2 model may take several minutes depending on the complexity of your data. It also requires sufficient RAM. Please be patient and allow enough time for this to run.
3. Once the spatial modelling is complete, the **map** and **datatable cards** will update with modelled data.
    - The map will update with the RR variable.
    - The map card will also update with a new map style dropdown on the right side of the card called **Border areas with excess risk dropdown** (Figure 13).
4. Select “Yes” on the **Border areas with excess risk dropdown**. The map will update with bordered areas specifically for those areas with an exceedance probability greater than 80% relative to the threshold selected in the **analytics sidebar menu**.
5. Use the slider in the **Exceedance probability** input to change the exceedance probability required for

bordering areas. For example, if the slider is set to 0.90, then areas with an exceedance probability equal or greater than 90% will be bordered.

**Figure 13.** Updating the map with bordered areas and the exceedance probability slider.

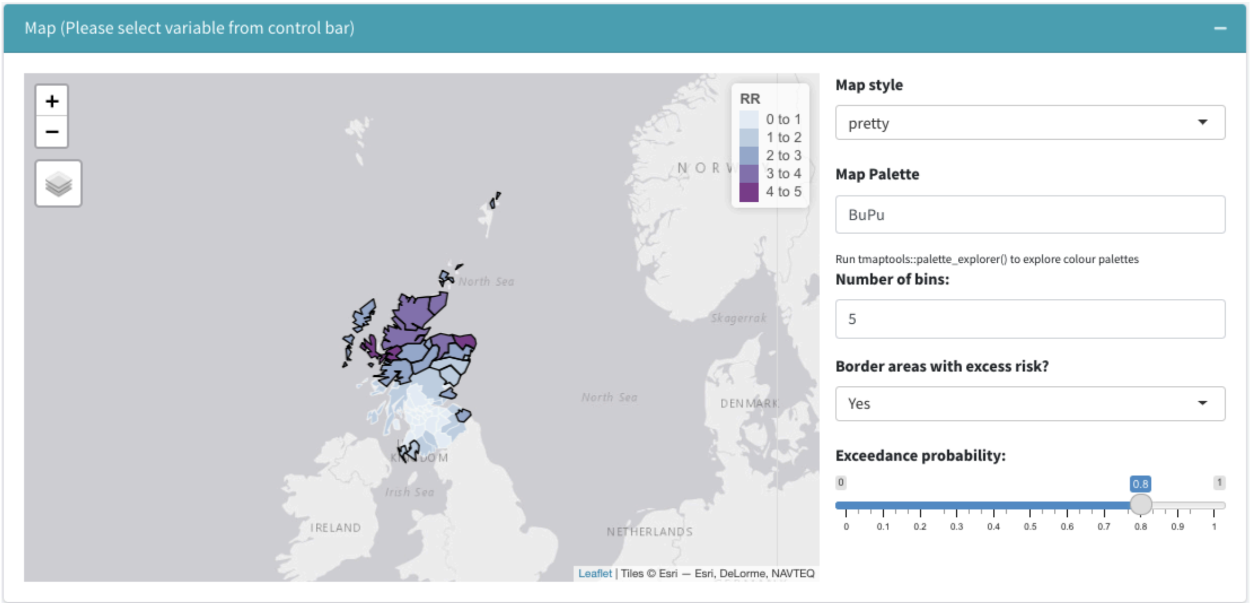


6. **Download model** results with the **button** on left navigation bar. This will download as an RData file, which can be loaded into R for further analysis (Figure 14).

**Figure 14.** Download Model data button.

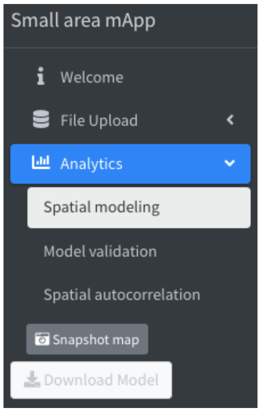


### *EXCESS CASES CARD*

1. The **"Excess cases among elevated areas" card** is on the bottom left of the main panel area. Click the "+" of the card (Figure 15).
    - NOTE: Areas deemed at an elevated risk are dependent on the relative risk threshold chosen in the **analytics sidebar menu**. As a default, this table identifies areas with an excess threshold of 80% or higher.

**Figure 15.** The Excess cases among elevated areas card.

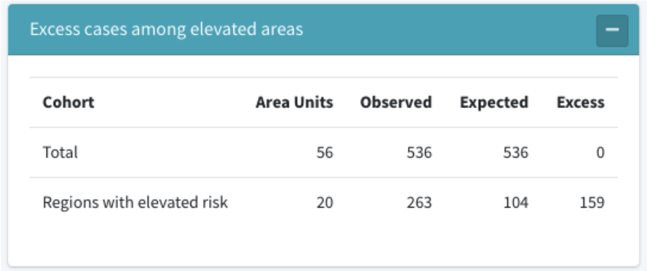
Excess cases among elevated areas

| Cohort | Area Units | Observed | Expected | Excess |
|---|---|---|---|---|
| Total | 56 | 536 | 536 | 0 |
| Regions with elevated risk | 20 | 263 | 104 | 159 |

### *SPATIAL STRUCTURED EFFECT CARD*

1. The **spatially structured effect card** is on the bottom right of the main panel area. Click the "+" to expand the card (Figure 16). The card presents a message with the proportion of the variation in the modelled relative risk that is explained by spatial correlations between neighbouring regions (the posterior median spatial random effect) and the corresponding 95% credible intervals.
    - NOTE: This card can only be used when spatial modelling is selected. When the card is expanded, a calculation on the back end is triggered. Therefore, this may take time to calculate and requires sufficient RAM. Please be patient and allow enough time for this to run.

**Figure 16.** The Spatial structured effect card.

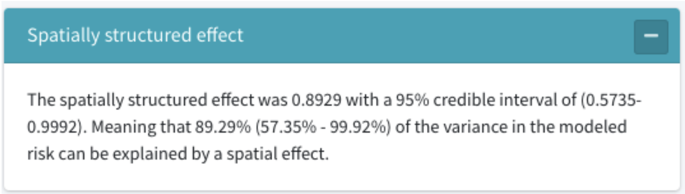


### MODEL VALIDATION SUBPAGE

1. In the **navigation panel,** click on the *model validation subpage.* This page contains two cards related to model validation – *Fitted vs. Observed values* and *Predictive integral transformed plot* (Figure 17).
2. The *Fitted vs. Observed values card* is built with the plotly R package and is interactive. Hover your mouse of data points to show corresponding data (e.g. SIRs, modelled RRs, area name). The R-squared value is in the bottom right corner of the plot.
3. You can take a snapshot by hovering your mouse over the plot and clicking the semi-transparent camera button on the top.
4. The *Predictive integral transformed plot card* is also built with the plotly R package and is interactive.

**Figure 17.** The model validation subpage.

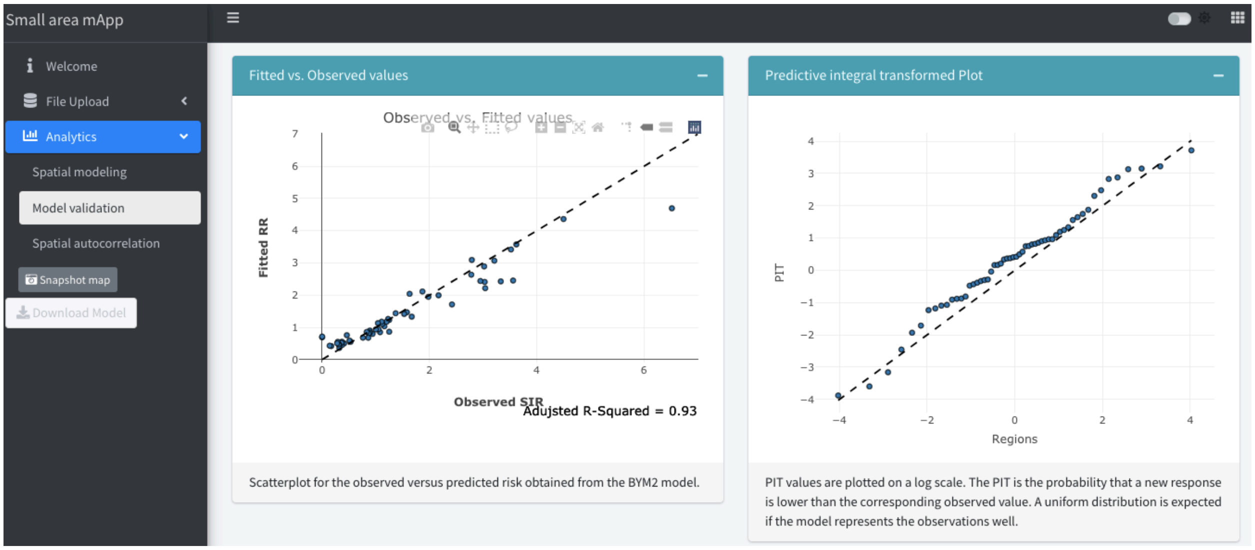


## SPATIAL AUTOCORRELATION SUBPAGE

1. In the **navigation panel,** click on the *Spatial autocorrelation subpage* (Figure 18). This page contains one card that runs a Global Moran's I test for spatial autocorrelation – *Moran's I test.* A Moran's I plot is generated with *ggplot* and includes the Moran's I statistic and associated p value in the bottom right. An figure legend is shown at the bottom of the card. This test is implemented by the spdep package, moran.mc() function.
    - NOTE: This test runs a monte carlo simulation and therefore, the results may change slightly on each run.

**Figure 18.** The model validation subpage.

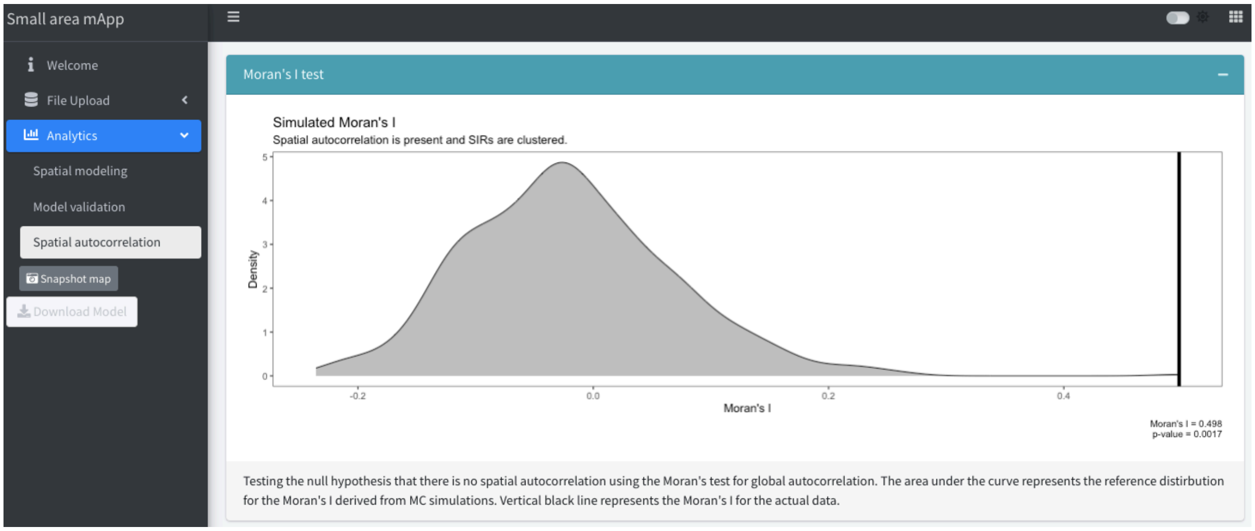

# REFERENCES


1. Martins TG, Simpson D, Lindgren F, Rue H. Bayesian computing with INLA: New features. Comput Stat Data Anal [Internet]. 2013;67:68–83. Available from: http://dx.doi.org/10.1016/j.csda.2013.04.014

2. Cressie NAC. Statistics for spatial data. Wiley; 1993. 573 p.

3. Clayton D, Kaldor J. Empirical Bayes Estimates of Age-Standardized Relative Risks for Use in Disease Mapping. Biometrics [Internet]. 1987 Sep;43(3):671–81. Available from: https://www.jstor.org/stable/2532003?origin=crossref
